# IL-27 promotes systolic overload-induced cardiac oxidative stress, inflammation, fibrosis, and heart failure development

**DOI:** 10.1016/j.redox.2026.104459

**Published:** 2026-10-28

**Authors:** Ziru Niu, Umesh Bhattarai, Dongzhi Wang, Xiaochen He, Lihong Pan, Heng Zeng, Jian-Xiong Chen, John S. Clemmer, Lei Hou, Yingjie Chen

**Affiliations:** a Department of Cardiology, Songjiang Hospital Affiliated to Shanghai Jiao Tong University School of Medicine, Shanghai, China; b Department of Physiology and Biophysics, School of Medicine, University of Mississippi Medical Center, Jackson, MS, United States; c Division of Clinical Pharmacology, Department of Medicine, Indiana University School of Medicine, Indianapolis, IN, United States; d Department of Pharmacology and Toxicology, School of Medicine, University of Mississippi Medical Center, Jackson, MS, United States

**Keywords:** IL-27, Inflammation, Heart failure

## Abstract

**Background:**

The interleukin-27 (IL-27) signaling axis plays a critical role in immunity and systemic metabolism. While it initiates potent pro-inflammatory responses against cancer and infection, heightened IL-27 activity during sterile inflammation can induce severe tissue damage. However, the specific function of IL-27 in regulating systolic overload-induced cardiac inflammation, oxidative stress, and heart failure (HF) pathogenesis remains unknown.

**Methods:**

We investigated the effects of genetic IL-27 receptor alpha (IL-27Rα) deficiency (IL-27Rα knockout), pharmacological IL-27 neutralization, and recombinant IL-27 administration on transverse aortic constriction (TAC)-induced HF in mice.

**Results:**

Cardiac IL-27 expression was significantly elevated in murine HF tissues. Global genetic ablation of IL-27Rα significantly suppressed TAC-induced cardiac inflammation, oxidative stress, fibrosis, hypertrophy, dysfunction, and mortality. Corroborating these protective effects, transcriptomic analysis (RNA-seq) revealed that collagen-containing extracellular matrix and extracellular regions were the top cellular components affected in IL-27Rα knockout mice following TAC. Specifically, IL-27Rα deficiency drastically suppressed pathways driving extracellular matrix remodeling, immune responses, and antigen presentation, while significantly downregulating networks governing systemic inflammation and oxidative stress. Furthermore, pharmacological neutralization of IL-27 effectively attenuated TAC-induced left ventricular (LV) leukocyte infiltration, oxidative stress, fibrosis, and dysfunction. Conversely, administration of recombinant mouse IL-27 exacerbated the TAC-induced cardiac accumulation of multiple immune cell subsets, oxidative stress, fibrosis, cardiomyocyte hypertrophy, and overall HF progression.

**Conclusions:**

Our findings demonstrate that IL-27 acts as a critical pathogenic driver of cardiac inflammation, oxidative stress, and HF development by modulating both immune cell activation and extracellular matrix remodeling following systolic overload.

## 1 Introduction

Heart failure (HF) is a progressive, leading global cause of morbidity and mortality [1], [2]. Despite diverse etiologies, chronic low-grade inflammation, metabolic dysfunction, and oxidative stress remain shared hallmarks of HF. Chronic systolic overload—often secondary to hypertension or aortic stenosis—increases left ventricular (LV) wall stress and represents a primary risk factor for HF [3], [4]. This sustained overload induces maladaptive remodeling, characterized by cardiomyocyte hypertrophy, immune cell accumulation, and fibrosis, ultimately culminating in HF [3], [4] alongside consequent pulmonary remodeling and right ventricular dysfunction [5], [6]. Recent studies demonstrate that chronic inflammation actively promotes HF pathogenesis, and its targeted inhibition effectively attenuates disease progression in experimental models [7], [8], [9]. Clinically, IL-1β blockade with canakinumab significantly reduced major cardiovascular events in patients with prior myocardial infarction or active inflammation [10], and dose-dependently decreased HF hospitalizations and related deaths [11]. However, the precise mechanisms by which inflammation drives HF remain incompletely understood.

Cardiac inflammation is driven by the dynamic interplay between innate and adaptive immunity [7], [8], a process orchestrated by cytokines, chemokines, their receptors, the initial tissue damage, and oxidative stress. Following initial cardiac injury, resident macrophages and recruited neutrophils initiate acute responses to clear cellular debris [8], [12]. Subsequently, infiltrating pro-inflammatory monocytes and other innate immune cells produce inflammatory mediators such as TNF-α and IL-1β [13], [14], [15]. Persistent cardiac stress further drives maladaptive remodeling, triggering antigen-presenting cell (APC)-dependent T cell activation in lymphatic tissues and subsequent cardiac T cell infiltration [16], [17], [18], [19]. Studies by our group and others demonstrate that monocyte-derived macrophages [13], CD8^+^ T cells [20], dendritic cells [21], NK1.1^+^ lymphocytes [22], and other immune subsets actively drive the overload-induced inflammatory microenvironment, oxidative stress, and pathological remodeling. Correspondingly, pro-inflammatory cytokines (IL-1β TNF-α) exacerbate this overload-induced inflammation, oxidative stress, fibrosis, and dysfunction, while targeted inhibition or anti-inflammatory mediators (e.g., IL-10), IL-35, neutralizing IL-1β or IL-12β, as well as antioxidants offer cardio-protection [15], [23], [24], [25], [26]. Furthermore, we found that blocking TAC-induced T cell activation—whether via genetic deletion of CD28 or CD80/CD86, or by inhibiting APC-T cell interactions with CTLA4-Ig—significantly attenuates LV hypertrophy and HF [18], [19]. Collectively, these findings highlight the essential pathogenic roles of macrophages, APCs, T cells, APC-T cell crosstalk, and pro-inflammatory cytokines in HF development.

The interleukin-27 (IL-27) signaling axis plays a critical role in immunity and systemic metabolism [27], [28], [29], [30], [31], [32]. Secreted primarily by activated antigen-presenting cells, the heterodimeric cytokine binds a receptor complex consisting of gp130 and the restricted IL-27Ra chain [33], [34], [35], which is highly expressed on naive T cells, monocytes, macrophages, dendritic cells, and non-immune cells [28], [34]. In immune cells, IL-27 receptor engagement activates JAK–STAT signaling, prominently involving STAT1 and STAT3, and can enhance IL-12 responsiveness in a cell context-dependent manner [28], [36], [37]. In naive CD4^+^ T cells, this axis directly upregulates the master transcription factor T-bet and the receptor subunit IL-12Rb2 [36], [37], [38], driving rapid differentiation and proliferation of Th1 effector cells [33], [38]. Concurrently, IL-27 also enhances CD8^+^ T-cell function and promotes the generation of cytotoxic T lymphocytes [39]. Moreover, IL-27 enhance oxidative stress through upregulation of ROS-generating machinery [40]. However, the effect of IL-27 signaling axis on modulating systolic overload-induced cardiac inflammation, oxidative stress, and HF development is still unknown.

In the present study, by using complementary genetic loss-of-function and pharmacological experimental approaches, we investigated the effects of IL-27 receptor knockout (IL-27Rα), IL-27 neutralizing antibodies, and the administration of recombinant IL-27 on transverse aortic constriction (TAC)-induced cardiac inflammation, cardiomyocyte hypertrophy, fibrosis, and left ventricular (LV) dysfunction in mice. We hypothesized that IL-27 signaling promotes pressure overload-induced HF by regulating myocardial inflammation, oxidative stress, and pathological remodeling.

## 2 Materials and methods

**Experimental Mice and Protocols** Wild-type (WT) C57BL/6J mice (Strain #000664) and IL-27Rα KO (Strain #018078) mice were purchased from The Jackson Laboratory. Mice were housed in a temperature-controlled environment with a 12-hour light/dark cycle and provided ad libitum access to food and water. For all cohorts, samples were collected when apparent LV dysfunction was observed in at least one of the experimental groups following TAC. Samples were harvested for subsequent flow cytometric, histological, immunohistological, and biochemical analyses. All procedures were approved by the Institutional Animal Care and Use Committee at the University of Mississippi Medical Center. *Detailed materials and methods are available in the online Data Supplement.*

**Pharmacological IL-27 Blockade and Recombinant IL-27 Administration** To evaluate the pharmacological inhibition of IL-27, WT male mice were subjected to TAC and randomly assigned to receive either an IL-27 neutralizing antibody (BioXCell, Catalog #BE0326) or an isotype control IgG. Treatments were administered via intraperitoneal injection at a dose of 250 μg/mouse every 3 days, starting 1-day post-TAC and continuing for a duration of 4 weeks. This dosage was adapted from a previous study [41], as it was effectively suppressed the acute graft-versus-host disease in mice. Samples for histological analysis were collected 4 weeks post-TAC. For gain-of-function studies, WT mice received recombinant murine IL-27 (rmIL-27; BioLegend, #577406) via intraperitoneal injection at a dose of 2.5 μg/mouse/day, or a saline vehicle, beginning approximately 1 h after TAC. The rmIL-27 dose was determined based on pilot dose-finding experiments with mild modification [29].

**Echocardiography** Echocardiography was performed using a VisualSonics Vevo 3100 imaging system (FUJIFILM VisualSonics Inc., Canada) at baseline and then at regular intervals (weekly for rmIL-27 cohorts and bi-weekly for antibody cohorts) post-TAC. Briefly, mice were anesthetized by inhalation of 1-2% isoflurane mixed with 100% oxygen. M-mode echocardiographic images were acquired and analyzed using Vevo LAB software (FUJIFILM VisualSonics Inc., Canada) to measure heart rate, LV ejection fraction, LV fractional shortening, LV end-systolic diameter, LV end-diastolic diameter, LV end-systolic volume, LV end-diastolic volume, LV anterior and posterior wall thickness at end-systole or end-diastole, stroke volume, and cardiac output. Doppler flow velocity in the right innominate artery and the left common carotid artery was measured using the pulsed-wave Doppler method.

**Flow Cytometry Analyses** For flow cytometric analysis of the lungs, tissues were perfused with cold PBS via the right ventricle and digested in HBSS containing collagenase D (1 mg/mL) at 37°C for 30 min using a Miltenyi tissue dissociator. Cell suspensions were filtered (100 μm), RBC-lysed, stained with a fixable viability dye, Fc-blocked with anti-CD16/32, and stained with fluorophore-conjugated surface antibodies. For intracellular cytokine assays, cells were stimulated with Cell Stimulation Cocktail plus Protein Transport Inhibitor Cocktail in RPMI-1640 + 10% FBS at 37°C for 2 h, followed by surface staining, permeabilization, and intracellular staining.

For LV flow cytometry, LV tissues were minced and digested in HBSS supplemented with DNase I (66.7 μg/mL) and Liberase (125 μg/mL) at 37°C for 30 min using a Miltenyi tissue dissociator. Staining and acquisition protocols were identical to those used for lung tissues. Immune subsets quantified included neutrophils, macrophages, CD11c^+^ dendritic cells, CD4^+^ T cells, and CD8^+^ T cells. Cytokine readouts focused on IL-1β and IFN-γ production, consistent with the key phenotypic endpoints. All data were acquired on a BD FACSymphony A3 and analyzed using FlowJo v10.

**Bulk RNA Sequencing** LV tissue samples were collected from WT and IL-27Rα KO female mice 4 weeks after TAC or sham surgery and stored at −80°C until RNA isolation. RNA integrity and quantity were assessed using the Agilent 2100 Bioanalyzer (Agilent Technologies, CA). High-quality RNA samples (RIN > 7) were submitted to Novogene Corporation (Sacramento, CA) for library construction and sequencing. The HiSeq 4000 platform was used to generate 150 bp paired-end reads. Raw reads were quality-checked and filtered, and clean reads were aligned to the mouse reference genome Mus_musculus_UCSC_mm39. Differential expression analysis was performed using the DESeq2 platform. Genes with an adjusted false discovery rate (FDR) < 0.05 were considered statistically significant differentially expressed genes.

**Statistical Analysis** Data are presented as mean ± SEM. For genotype-based comparisons under sham vs. TAC conditions, a two-way ANOVA followed by a Bonferroni post hoc test was used. For two-group comparisons, an unpaired *t*-test was utilized. Survival curves were analyzed using the log-rank test. For pharmacological studies involving three groups, a one-way ANOVA with a Bonferroni post hoc test was applied. All statistical analyses were performed using GraphPad Prism 10, and a p-value < 0.05 was considered statistically significant.

## 3 Results

### 3.1 Cardiac IL-27 expression is increased after HF

To investigate the association between IL-27 and heart failure, the expression of IL-27p28 (the unique subunit of IL-27) was evaluated in wild-type mice after TAC. TAC-induced pressure overload resulted in a significant elevation of cardiac IL-27p28. In addition, TAC caused a significant increase in cardiac MCP-1 (monocyte chemoattractant protein-1) and IL-1α protein levels (Supplemental Fig. S1).

### 3.2 Deletion of IL-27Rα improves survival and preserves ventricular function following pressure overload

To evaluate the *in vivo* pathophysiological role of the IL-27/IL-27Rα axis during heart failure development, we subjected both male and female WT and IL-27Rα KO mice to pressure overload via TAC. The experimental design incorporated baseline echocardiography followed by serial ultrasound monitoring at 2, 4, and 6 weeks post-surgery in male mice (Fig. 1A). *Pulsed-wave Doppler measurements show that TAC resulted in comparable degrees of aortic constriction in WT and IL-27Rα KO mice as evidenceed by similar pre-to post-constriction flow velocity ratios (*Supplemental Fig. S2*).* Over a 6-week monitoring period, male IL-27Rα KO mice exhibited a striking survival advantage compared to WT controls (P = 0.0111; Fig. 1B), a protective effect that occurred independently of any significant divergence in body weight trajectories (Fig. 1C). Serial echocardiographic assessments (Fig. 1D) revealed severe, progressive cardiac dysfunction in WT males following TAC. In contrast, IL-27Rα deficiency robustly preserved systolic function and cardiac geometry, evidenced by a significantly higher left ventricular ejection fraction (LVEF) and attenuated expansion of both LV end-systolic and end-diastolic diameters throughout the 6-week time course (Fig. 1E; Supplemental Table S3).

**Fig. 1:**
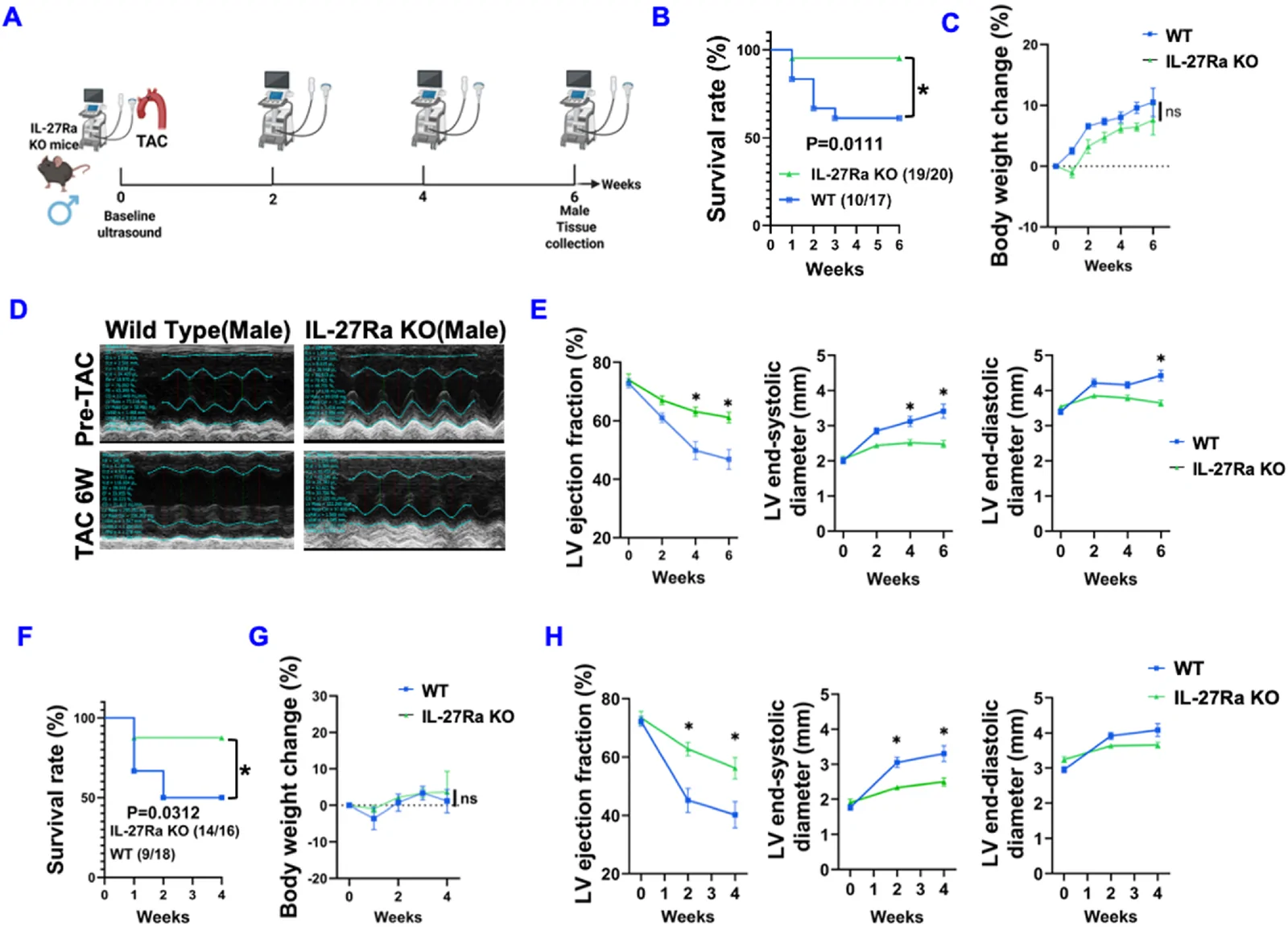
**IL-27Rα deletion protects against TAC-induced mortality and cardiac dysfunction in male and female mice. (A)** Schematic of the experimental timeline detailing baseline echocardiography, transverse aortic constriction (TAC) surgery, and subsequent monitoring up to 6 weeks in male cohorts. **(B)** Kaplan-Meier survival curve demonstrating a significantly higher survival rate in male IL-27Rα KO mice (green) compared to WT controls (blue) following TAC (P = 0.0111). **(C)** Time-course analysis of body weight changes (%) in male WT and IL-27Rα KO mice following TAC (ns = not significant). **(D, E)** Representative M-mode echocardiograms **(D)** and time-course quantification **(E)** of left ventricular ejection fraction (LVEF), LV end-systolic diameter, and LV end-diastolic diameter in males. KO mice maintained significantly higher LVEF and attenuated pathogenic LV dilation post-TAC. **(F)** Kaplan-Meier survival curve for the female cohort over 4 weeks post-TAC (P = 0.0312). **(G)** Corresponding body weight changes (%) for the female cohort post-TAC (ns = not significant). **(H)** Time-course quantification of corresponding echocardiographic parameters (LVEF, LV end-systolic diameter, and LV end-diastolic diameter) in females. Male WT: n = 10/17 (survived/total); Male IL-27Rα KO: n = 19/20. Female WT: n = 9/18; Female IL-27Rα KO: n = 14/16. Data are presented as mean ± SEM. *p < 0.05.

The macroscopic cardioprotective effects were conserved across sexes. Female IL-27Rα KO mice similarly demonstrated significantly reduced mortality (P = 0.0312; Fig. 1F) and stable body weight (Fig. 1G) over a 4-week post-TAC period compared to their WT counterparts. Corresponding echocardiographic analysis in the female cohort confirmed that the deletion of IL-27Rα fundamentally protected against TAC-induced LVEF decline and progressive left ventricular dilation (Fig. 1H; Supplemental Table S5). Together, these baseline physiological data establish that intact IL-27Rα signaling actively drives pressure overload-induced mortality and the progressive deterioration of ventricular systolic function.

### 3.3 IL-27Rα deficiency blunts pathological cardiac hypertrophy and secondary pulmonary congestion

Consistent with the preservation of ventricular systolic function, the absence of intact IL-27 signaling markedly attenuated macroscopic pathological remodeling and the physical signs of congestive heart failure. Morphometric tissue analysis in the male cohorts revealed that while pressure overload provoked profound, multi-chamber cardiac hypertrophy in WT mice—evidenced by massive increases in both left ventricular (LV) and left atrial (LA) weight-to-tibial length ratios (Fig. 2A and B, Supplemental Table S2)—this structural hypertrophic response was robustly blunted in IL-27Rα KO mice. Moreover, IL-27Rα KO significantly attenuated TAC-induced cardiomyocyte hypertrophy in male mice(Supplemental Fig. S3). Furthermore, WT males exhibited severe pulmonary congestion and compensatory right ventricular (RV) remodeling following TAC. In striking contrast, IL-27Rα deletion effectively normalized lung and RV weights, mitigating these downstream physical sequelae of left-sided heart failure (Fig. 2C, D, Table S2).

**Fig. 2:**
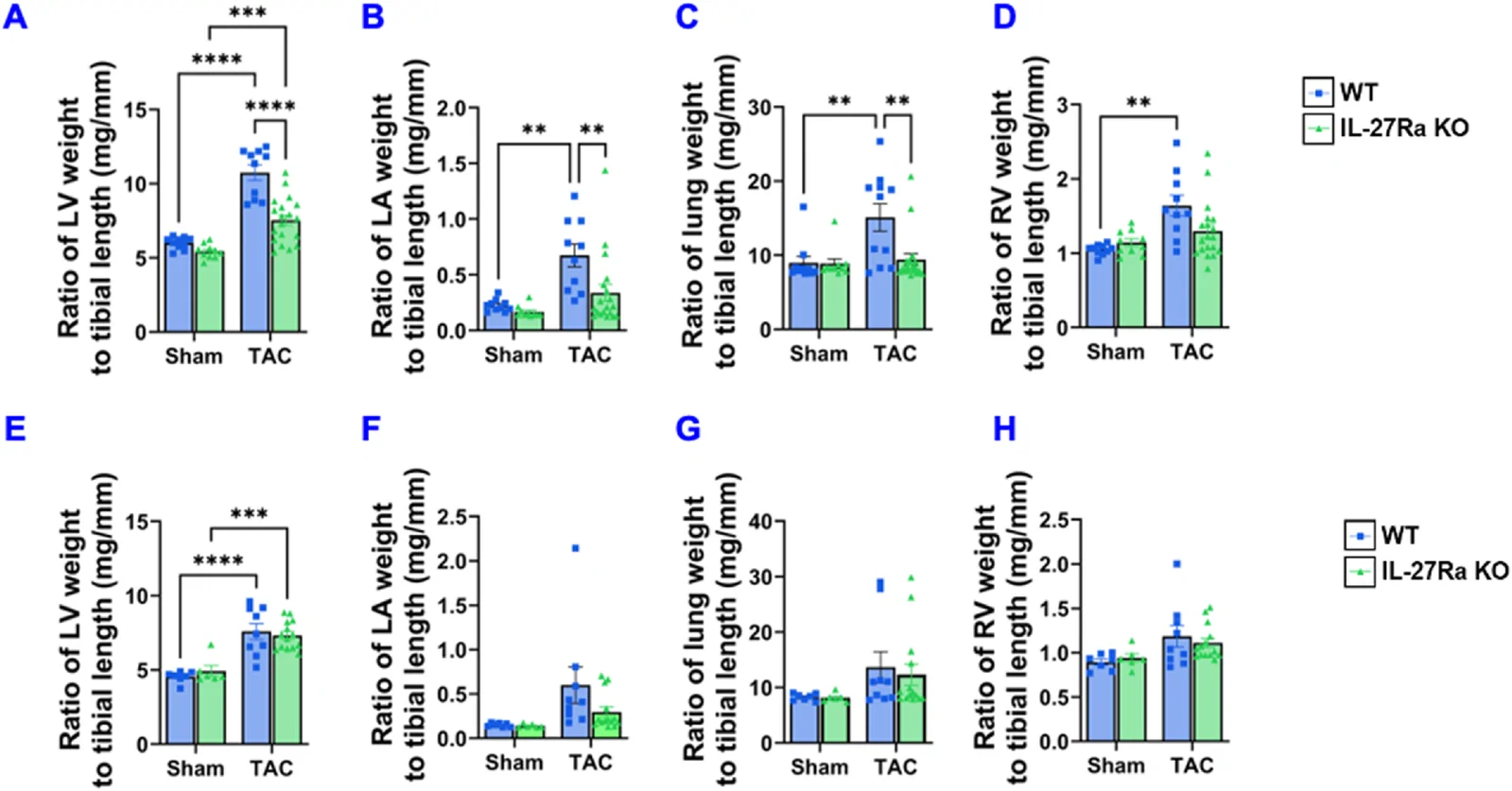
**Effect of IL-27Rα deletion on TAC-induced cardiac hypertrophy and pulmonary congestion. (A–D)** Morphometric quantification of **(A)** left ventricular (LV), **(B)** left atrial (LA), **(C)** lung, and **(D)** right ventricular (RV) weights normalized to tibial length (mg/mm) in male wild-type (WT, blue) and IL-27Rα KO (green) mice subjected to Sham or transverse aortic constriction (TAC) surgery. The data demonstrate significantly reduced pathological LV and LA remodeling, alongside a mitigation of pulmonary congestion and secondary RV hypertrophy in KO males following TAC. **(E–H)** Corresponding morphometric analysis of **(E)** LV, **(F)** LA, **(G)** lung, and **(H)** RV weight-to-tibial length ratios in female WT and IL-27Rα KO mice following Sham or TAC surgery. While TAC induced significant LV hypertrophy in both WT and KO females compared to their respective sham controls, no statistical difference was observed between the WT and KO groups following TAC at the study endpoint in female mice. Male WT: n = 10; Male IL-27Rα KO: n = 19. Female WT: n = 9; Female IL-27Rα KO: n = 14. Data are presented as mean ± SEM. **p < 0.01, ***p < 0.001, and ****p < 0.0001.

In the female cohorts, TAC caused significant LV hypertrophy in both WT and KO mice as compared with their corresponding sham groups (Fig. 2E–Table S4). However, there was no statistical difference in LV hypertrophy, nor in LA, lung, and RV expansion, between the WT and KO mice following TAC at the end of the study (Fig. 2E–H, Table S4). This lack of statistical divergence between the post-TAC genotypes is likely a reflection of survivorship bias. Because morphometric tissues were collected exclusively at the study's endpoint, the analysis inherently excludes the approximately 50% of WT TAC mice that succumbed to premature mortality. Given that these non-surviving WT mice presumably experienced the most severe, fatal degrees of structural remodeling and pulmonary edema, the surviving WT cohort represents a self-selected group with a milder disease phenotype. Consequently, this end-point tissue evaluation likely underestimates the true physiological magnitude by which IL-27Rα deletion protects against catastrophic pathological remodeling and systemic congestion.

### 3.4 IL-27Rα deficiency globally alters the cardiac transcriptome, suppressing pathogenic remodeling and enhancing metabolic networks

To delineate the downstream molecular mechanisms by which IL-27Rα deletion protects against pressure overload, bulk RNA-sequencing was performed on left ventricular tissues from female WT and IL-27Rα KO mice. Principal component analysis (PCA) and hierarchical clustering of the transcriptomic data demonstrated highly distinct gene expression profiles among the groups. Notably, the WT TAC group clustered entirely separately from the sham controls, whereas the KO TAC group exhibited an intermediate transcriptional signature that remained much closer to the baseline control state (Fig. 3A and B). Differential gene expression analysis comparing KO TAC to WT TAC tissues, visualized via volcano plot, revealed a widespread downregulation of numerous pathological and profibrotic transcripts in the KO group, prominently including genes such as *Postn*, *Cilp*, *Col12a1*, *Col8a2*, and *Myh7* (Fig. 3C). KEGG analysis confirmed that the major gene expression differences were heavily concentrated in pathogenic disease networks, including dilated and hypertrophic cardiomyopathy pathways, as well as extracellular matrix (ECM)-receptor interactions (Fig. 3D). Gene Ontology (GO) analyses of these differentially expressed genes highlighted a dominant representation of terms associated with the extracellular matrix (ECM), including “collagen-containing extracellular matrix”, “extracellular region”, “extracellular matrix”, “basement membrane,” and “collagen trimer” organization (Fig. 3E).

**Fig. 3:**
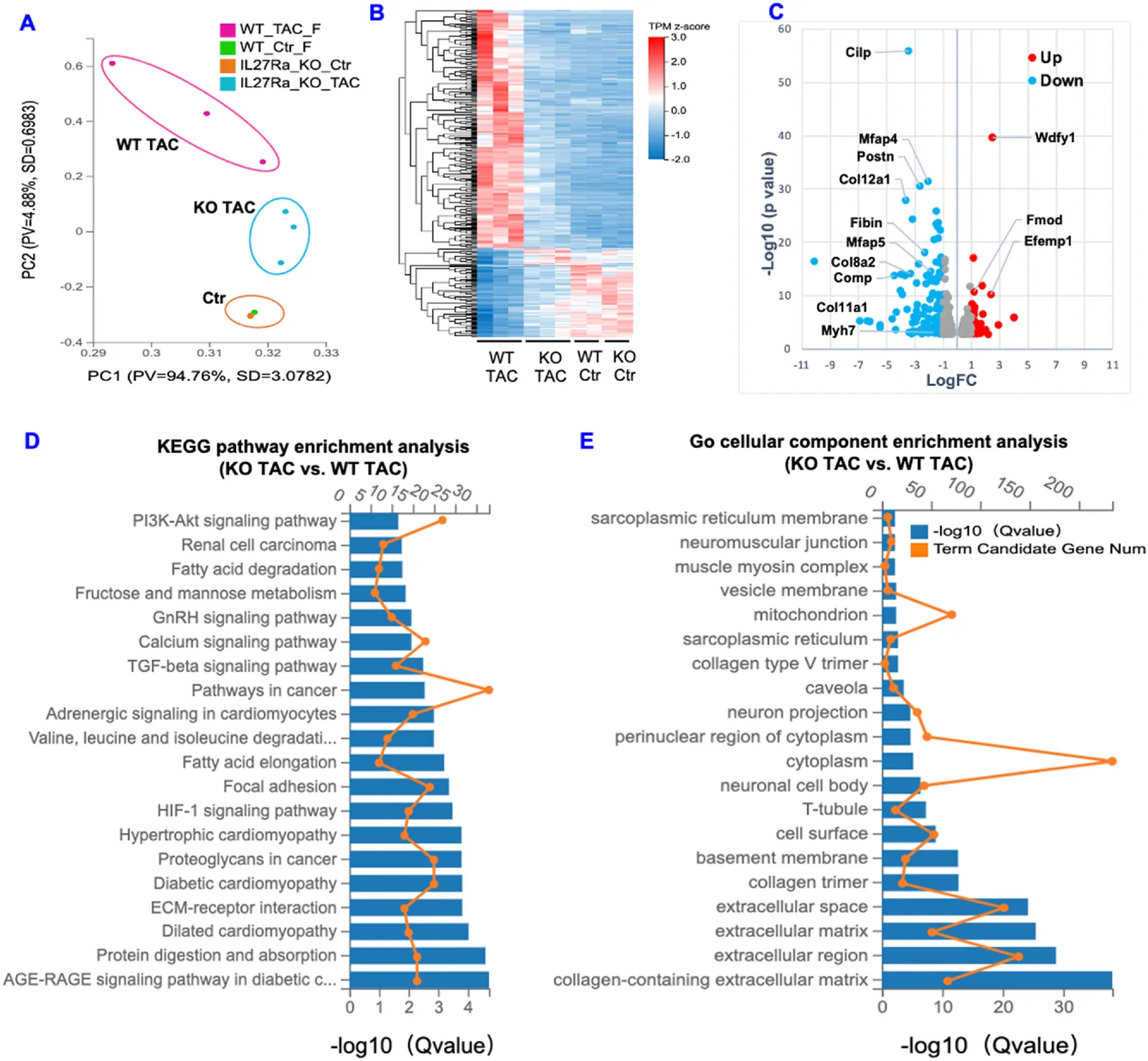
**IL-27Rα deletion blunts TAC-induced transcriptomic alterations and suppresses fibrotic gene networks. (A, B)** Principal component analysis (PCA) plot **(A)** and hierarchical clustering heatmap **(B)** of bulk RNA-seq data from female left ventricular tissues. The IL-27Rα KO TAC cohort exhibits an intermediate transcriptomic shift compared to WT TAC and Sham (Ctr) controls. **(C)** Volcano plot of differentially expressed genes (KO TAC vs. WT TAC). Blue dots denote significantly downregulated genes (e.g., Postn, Cilp, Col12a1, Myh7); red dots indicate upregulated genes. **(D)** KEGG pathway analysis (KO TAC vs. WT TAC) highlighting the significant modulation of disease pathways, including cardiomyopathy and ECM-receptor interactions. **(E)** Gene Ontology (GO) cellular component enrichment analysis demonstrating that the top downregulated networks in KO TAC mice are heavily associated with extracellular matrix remodeling and collagen structures. *TAC: n = 3 per group; Control group (Ctr): n = 2 per group.*

To identify the core biological pathways altered in this model, we performed Gene Set Enrichment pathway analysis (Fig. 4A and B). The analysis revealed a distinct dichotomy between upregulated and downregulated gene sets. Pathways exhibiting positive enrichment were overwhelmingly associated with mitochondrial and cellular metabolism, with the most significant upregulation seen in amino acid degradation (valine, leucine, and isoleucine), fatty acid degradation, and the citrate (TCA) cycle (Fig. 4B, Supplemental Fig. S4A and S4B). Conversely, pathways exhibiting negative enrichment scores were heavily dominated by ECM remodeling, immune and inflammatory responses, and infectious disease pathways (Fig. 4A, Supplemental Fig. S4C). Notably, ECM-receptor interaction (Fig. 4A,C) and various pathogen-response pathways showed the most significant downregulation, highlighting a broad suppression of specific structural and inflammatory signaling cascades alongside the enhancement of metabolic function.

**Fig. 4:**
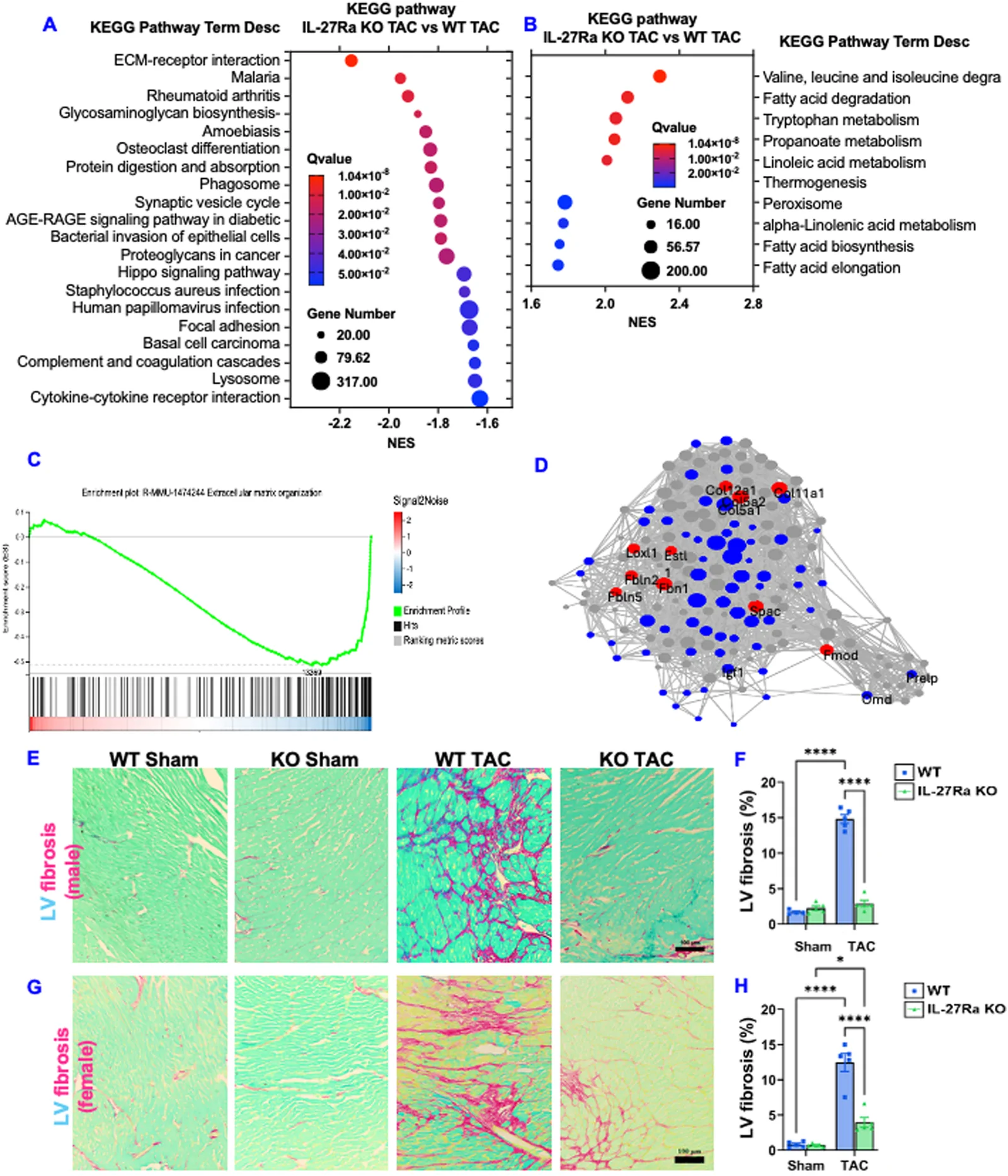
**IL-27Rα deletion suppresses pro-fibrotic gene networks and attenuates TAC-induced myocardial fibrosis. (A, B)** Bubble plots displaying the top significantly down-regulated **(A)** and up-regulated **(B)** GSEA KEGG pathways in the myocardium of IL-27Rα KO mice following TAC compared to WT TAC controls. **(C)** Representative GSEA plot demonstrating the significant downregulation of the extracellular matrix organization pathway in the IL-27Rα KO TAC cohort. **(D)** Key Driver Analysis (KDA) network mapped from the differential transcriptomic data. Red nodes represent the principal key driver genes (e.g., *Col12a1*, *Col11a1*, *Col5a1*, *Fbln2*, *Loxl1*) orchestrating the network, and blue nodes represent the selected associated network genes. **(E–H)** Representative histological images of left ventricular (LV) tissue stained for fibrosis (collagen deposition in pink/red) and corresponding quantitative bar graphs in male **(E, F)** and female **(G, H)** mice. The data demonstrate a significant reduction in LV fibrosis (%) in both male and female IL-27Rα KO mice compared to WT controls following TAC. *(****E–H***): n= 4-5 per group. Data are presented as mean ± SEM. Statistical significance is denoted by * p < 0.05 and ****p < 0.0001.

### 3.5 Key Driver Analysis confirms a mechanism of attenuated cardiac fibrosis

To identify the central regulatory nodes driving these distinct transcriptomic responses in IL-27Rα KO mice during pressure overload, we performed a Key Driver Analysis (KDA) on the bulk cardiac RNA-seq data. KDA analysis was built according to the significantly DEGs, and the result revealed a tightly regulated network associated with ECM organization, where critical structural and cross-linking genes—such as *Fbn1*, *Loxl1*, *Col12a1*, and *Fmod*—act as Key Drivers (Fig. 4D, red nodes) that dictate the broader downregulation of selected structural genes (blue nodes), including prominent fibrotic markers like *Fn1* and *Col1a1*. This architecture reaffirms that the loss of IL-27Rα actively blunts the conventional fibrotic response and ECM deposition typically induced by TAC.

### 3.6 Histological validation confirms attenuated myocardial fibrosis in IL-27Rα deficient mice

To validate these transcriptomic predictions *in vivo*, we evaluated myocardial interstitial fibrosis histologically at the study endpoint. In perfect alignment with the profound suppression of fibrotic gene networks, IL-27Rα KO mice were highly protected against TAC-induced collagen deposition. Both male (Fig. 4E and F) and female (Fig. 4G and H) knockout cohorts exhibited a striking, statistically significant reduction in left ventricular fibrotic area following TAC compared to their heavily fibrotic WT counterparts. Together, these integrated transcriptomic and histological findings confirm that the IL-27/IL-27Rα axis is a requisite driver of the pathological extracellular matrix remodeling underlying heart failure progression.

### 3.7 Identification of significantly changed genes associated with immunity and inflammation in IL-27Ra KO mice

Functional classification of differentially expressed genes revealed a robust enrichment in pathways related to the “Immune system” and infectious diseases (viral, bacterial, and parasitic) (Supplemental Fig. S5A). Consistent with this functional classification, hierarchical clustering and expression heatmap analysis identified a distinct module of highly upregulated genes intricately linked to immune activation, inflammation, and pathogen response (Supplemental Fig. S5B). Among the top upregulated transcripts in this dominant cluster were several critical components of the complement system (***C4b***, ***C7***, ***Cfh***, and ***Serping1***), which play a vital role in innate immunity and inflammation. Furthermore, the analysis revealed prominent upregulation of ***Lbp*** (Lipopolysaccharide binding protein), a classic acute-phase protein involved in the immune response to bacterial infection; ***Spp1*** (Osteopontin), a key pro-inflammatory and pro-fibrotic cytokine; the chemokine ***Ccl21a***, which directs immune cell migration; and ***Cd44***, a critical surface receptor mediating leukocyte activation and adhesion. Paradoxically, *Wdfy1,* a pro-inflammatory adapter for TLR3/TLR4 signaling, was the top upregulated gene in KO mice after TAC (Fig. S5B, Fig. 3C). Together, these transcriptomic signatures confirm a widespread and potent upregulation of networks governing innate immunity and inflammatory signaling.

### 3.8 IL-27Rα deficiency attenuates myocardial inflammation and oxidative stress

To investigate the impact of IL-27Rα deletion on the local immune response during pressure overload, we evaluated myocardial leukocyte infiltration. Immunofluorescence staining revealed a massive influx of CD45^+^ leukocytes in the left ventricles of WT mice following TAC. In striking contrast, this severe inflammatory infiltration was significantly blunted in both male (Fig. 5A and B) and female (Fig. 5C and D) IL-27Rα KO mice, indicating a sex-independent mechanism by which IL-27 signaling promotes widespread immune cell recruitment to the stressed heart. To further characterize this local immune response, we performed flow cytometric profiling of the LV immune cell subsets (Fig. 5E). The analysis demonstrated that the TAC-induced expansion of the macrophage (Mp) population observed in WT mice was significantly suppressed in the IL-27Rα knockout cohort, indicating a fundamental modulation of the innate immune response to hemodynamic stress. Collectively, these data suggest that intact IL-27 signaling is critical not only for driving total leukocyte recruitment but also for orchestrating the specific, macrophage-heavy inflammatory microenvironment typical of heart failure.

**Fig. 5:**
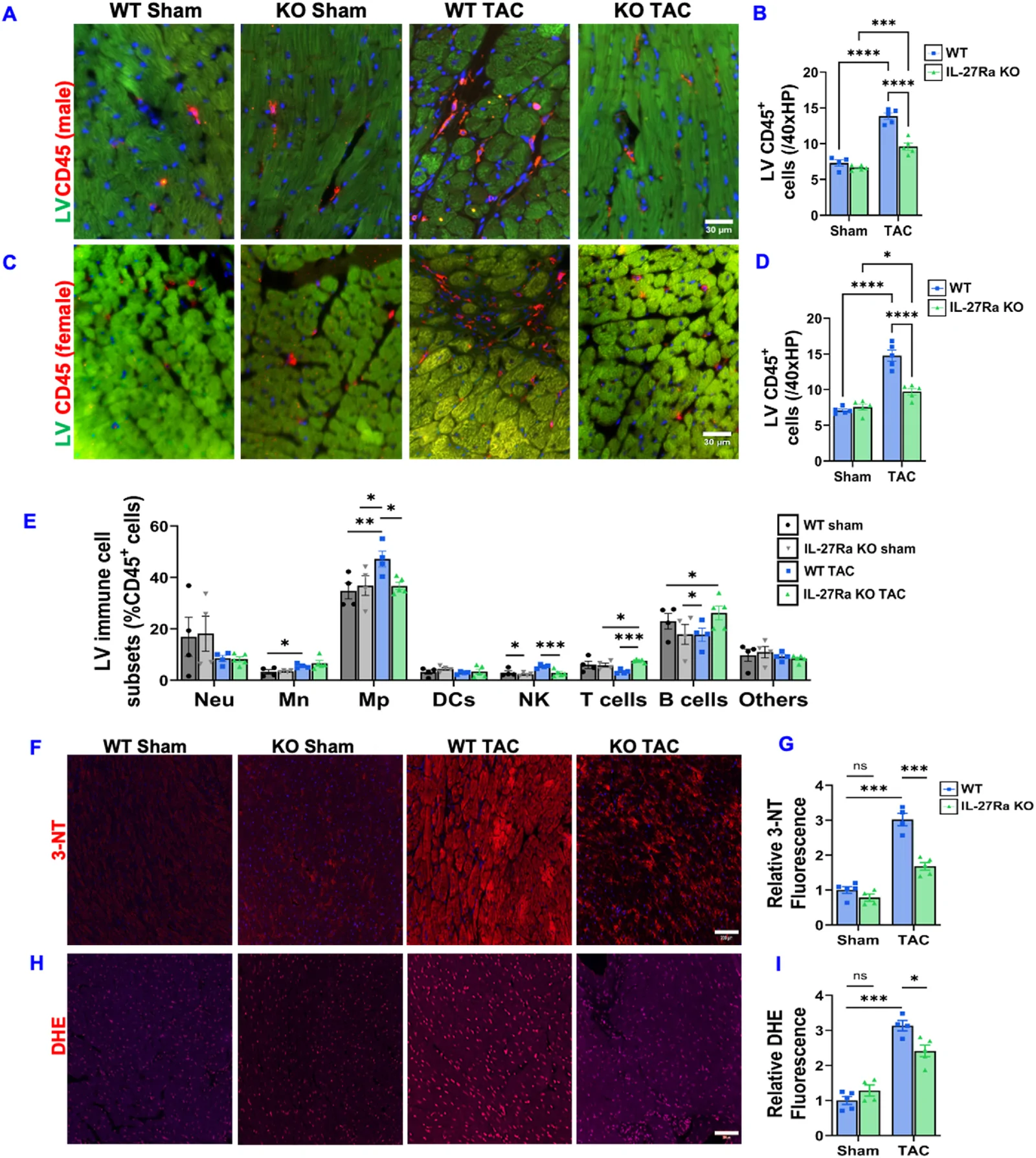
***IL-27Rα deficiency blunts TAC-induced myocardial leukocyte infiltration, modulates immune cell composition, and attenuates oxidative stress.(A–D)*** Representative immunofluorescence images and quantification of CD45^+^ leukocytes in the left ventricle (LV) of male **(A, B)** and female **(C, D)** mice. IL-27Rα KO mice exhibit a significantly reduced accumulation of CD45^+^ cells post-TAC compared to WT controls across both sexes. **(E)** Flow cytometric profiling of LV immune cell subsets in male mice, highlighting a significant suppression of TAC-induced macrophage (Mp) expansion in the KO cohort. **(F–I)** Representative images and quantification of left ventricular 3-nitrotyrosine (3-NT) **(F, G)** and dihydroethidium (DHE) **(H, I)** staining. The data confirm profoundly attenuated nitrosative and oxidative stress in IL-27Rα KO mice following TAC compared to WT controls. n = 4-5 per group. Data are presented as mean ± SEM. Statistical significance is denoted by * p < 0.05, ns = not significant.

Given the well-established interplay between myocardial inflammation and ROS generation in the pathogenesis of heart failure, we next assessed cardiac oxidative and nitrosative stress. WT mice exhibited severe oxidative damage post-TAC, as evidenced by intense 3-nitrotyrosine (3-NT) accumulation (Fig. 5F and G) and highly elevated dihydroethidium (DHE) oxidation (Fig. 5H and I). Consistent with their reduced inflammatory burden, IL-27Rα KO mice displayed profoundly attenuated levels of both 3-NT and DHE fluorescence. Collectively, these findings demonstrate that the IL-27/IL-27Rα axis actively promotes pathogenic myocardial leukocyte recruitment—particularly macrophage accumulation—and drives localized oxidative stress during chronic hemodynamic overload.

### 3.9 IL-27Rα deficiency protects against secondary pulmonary inflammation, fibrosis, and vascular remodeling

Left-sided heart failure induced by chronic pressure overload frequently leads to severe secondary pulmonary complications, including congestion and structural remodeling. Given that lung weight was significantly increased in male mice after TAC, we next evaluated whether IL-27Rα deletion protected the pulmonary microenvironment from these systemic sequelae by assessing inflammation, structural fibrosis, and vessel muscularity at the study endpoint.

Immunofluorescence staining of lung tissue sections revealed a massive accumulation of CD45^+^ leukocytes in WT mice following TAC, an inflammatory response that was significantly blunted in IL-27Rα KO mice (Fig. 6A and B). Furthermore, histological evaluation demonstrated that TAC provoked significant interstitial fibrosis and vascular remodeling in the lungs of WT controls. In striking contrast, IL-27Rα deficiency robustly protected against these structural alterations, showing a dramatic, statistically significant reduction in lung fibrotic area (Fig. 6C and D) and completely preventing the abnormal increase in fully muscularized pulmonary arterioles observed post-TAC (Fig. 6E and F). Together, these findings indicate that targeting the IL-27/IL-27Rα axis effectively shields the pulmonary system from the secondary inflammatory, fibrotic, and vascular consequences of pressure overload-induced heart failure.

**Fig. 6:**
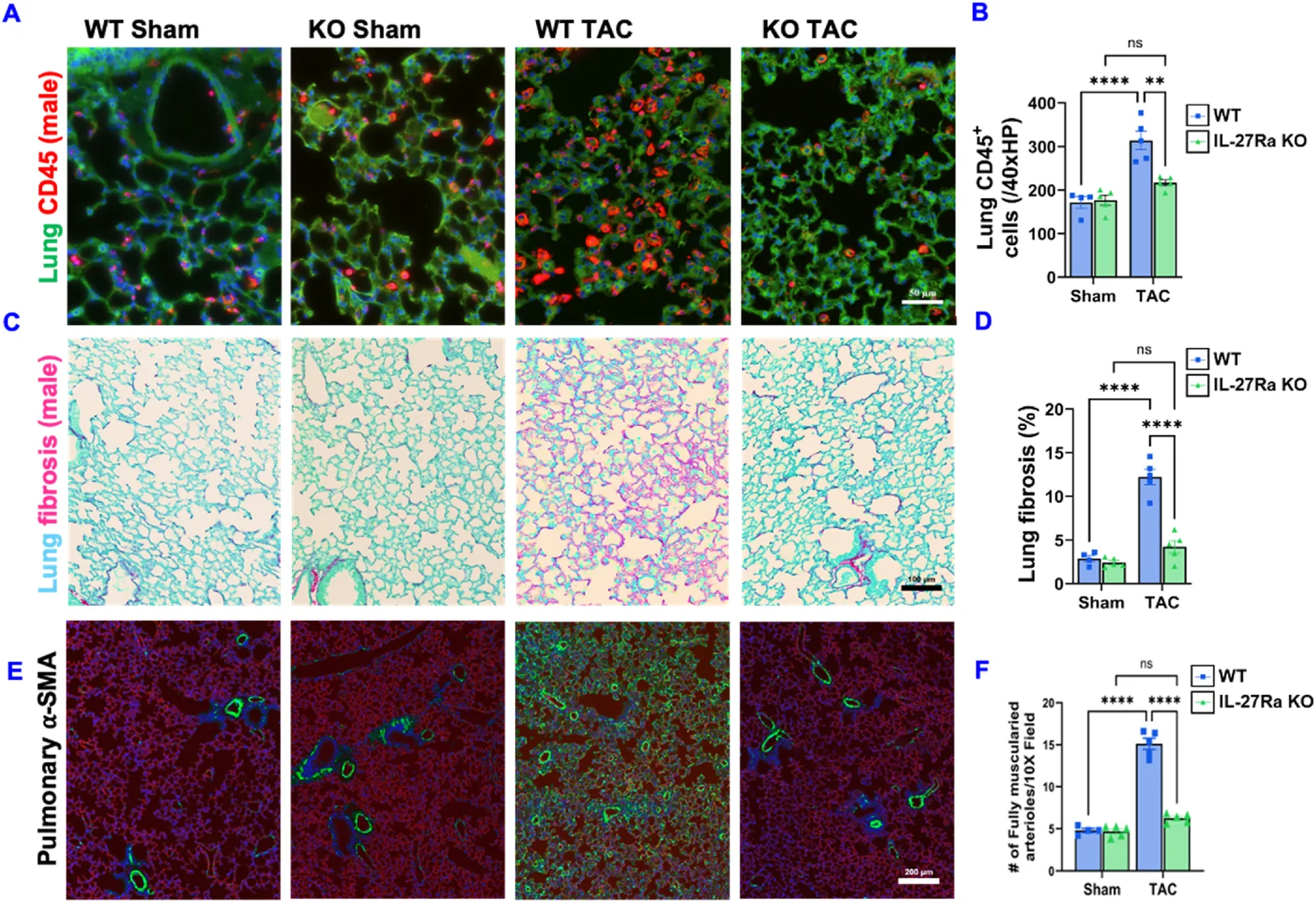
**IL-27Rα deletion attenuates TAC-induced secondary pulmonary inflammation, fibrosis, and vascular remodeling in male mice. (A, B)** Representative immunofluorescence images **(A)** and quantification **(B)** of CD45^+^ leukocytes (red) in lung sections from male wild-type (WT) and IL-27Rα KO mice subjected to Sham or TAC surgery. Nuclei are counterstained with DAPI (blue). IL-27Rα KO mice exhibit a significantly blunted accumulation of CD45^+^ cells post-TAC compared to WT controls. **(C, D)** Representative Sirius Red-stained lung sections **(C)** and quantitative bar graph **(D)** demonstrating a significant reduction in pulmonary fibrotic area (%) in IL-27Rα KO mice following TAC compared to WT controls. **(E, F)** Representative immunofluorescence images **(E)** and quantification **(F)** of fully muscularized pulmonary arterioles per 10X field. Pressure overload provoked a dramatic increase in arteriole muscularity in WT mice, whereas IL-27Rα deletion completely prevented this vascular remodeling. n = 5/group. Data are presented as mean ± SEM. **p < 0.01 and ****p < 0.0001; ns = not significant.

### 3.10 Therapeutic neutralization of IL-27 preserves cardiac function and blunts pathological remodeling following pressure overload

Having established that genetic ablation of the IL-27 receptor profoundly protects against heart failure, we next sought to determine the translational therapeutic potential of targeting this axis pharmacologically. To this end, we evaluated whether direct blockade of the IL-27 ligand could confer comparable cardiac protection in wild-type mice subjected to acute pressure overload. Following TAC surgery, mice were treated with either an IL-27 neutralizing monoclonal antibody (IL-27 mAb) or an IgG isotype control via regular intraperitoneal injections over a 4-week period (Fig. 7A). *Pulsed-wave Doppler measurements show that TAC resulted in comparable degrees of aortic constriction in IgG- and IL-*27 mAb*-treated mice* (Supplemental Fig. S6).

**Fig. 7:**
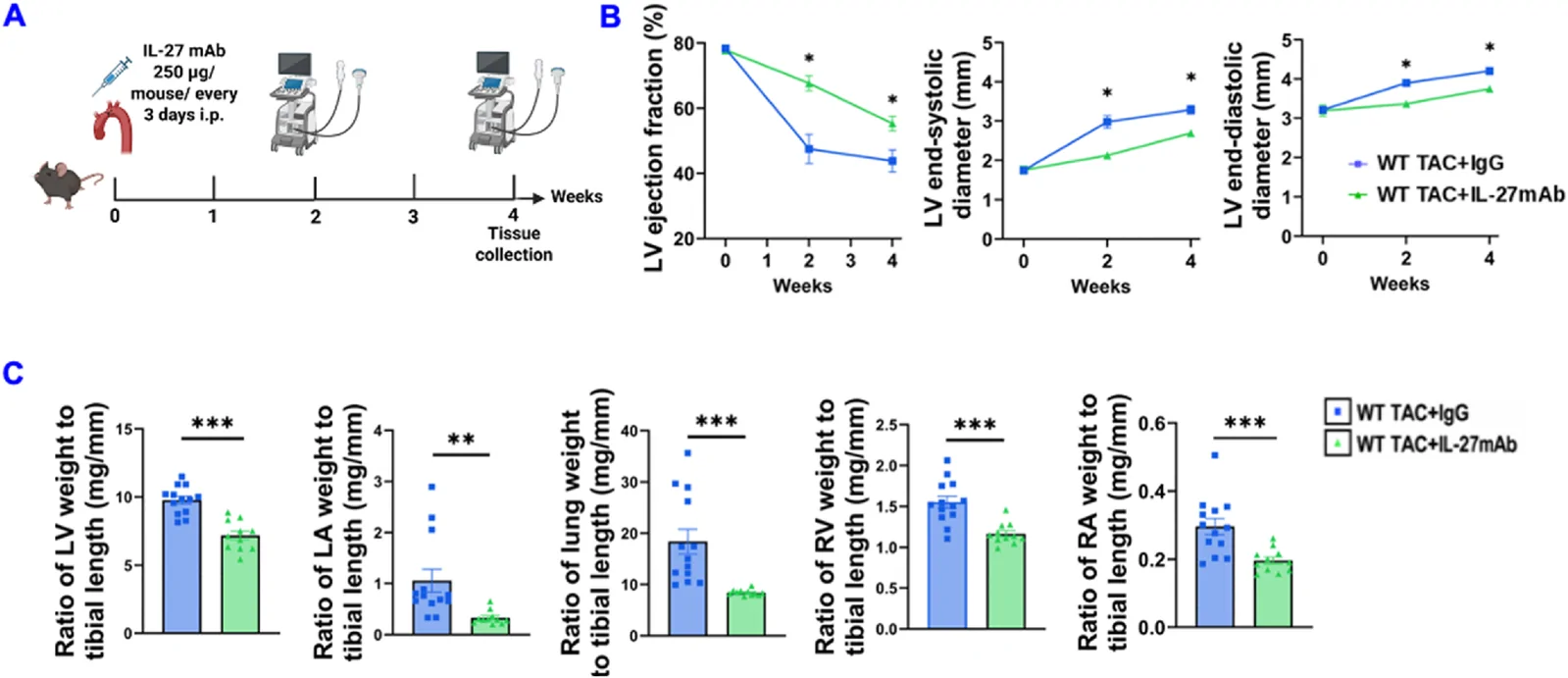
**Pharmacological neutralization of IL-27 attenuates TAC-induced cardiac dysfunction and multi-chamber hypertrophy. (A)** Schematic representation of the experimental design. Following transverse aortic constriction (TAC) surgery, wild-type mice were administered either an IL-27 neutralizing monoclonal antibody (IL-27 mAb; 250 μg/mouse every 3 days i.p.) or an IgG isotype control for 4 weeks. **(B)** Time-course quantification of longitudinal echocardiographic parameters, including left ventricular ejection fraction (LVEF), LV end-systolic diameter, and LV end-diastolic diameter. Pharmacological blockade with IL-27 mAb significantly preserved LVEF and attenuated pathogenic LV chamber dilation at 2 and 4 weeks post-TAC compared to the IgG control cohort. **(C)** Morphometric quantification of left ventricular (LV), left atrial (LA), lung, right ventricular (RV), and right atrial (RA) weights normalized to tibial length (mg/mm) at 4 weeks post-TAC. Therapeutic neutralization of IL-27 significantly blunted pathological multi-chamber structural remodeling and secondary pulmonary congestion. *n = 13-14 per group*. Data are presented as mean ± SEM. Statistical significance is denoted by * p < 0.05, **p < 0.01, and ***p < 0.001.

Longitudinal echocardiographic monitoring revealed that therapeutic neutralization of IL-27 robustly preserved ventricular systolic function. Compared to the rapid, progressive functional deterioration observed in the IgG control cohort, mice treated with the IL-27 mAb maintained a significantly higher LVEF and exhibited markedly attenuated left ventricular chamber dilation (end-systolic and end-diastolic diameters) at both 2 and 4 weeks post-TAC (Fig. 7B; Table S7).

Consistent with the preservation of *in vivo* cardiac geometry and contractility, post-mortem gravimetric analysis at the 4-week endpoint confirmed profound structural protection at the tissue level. Treatment with the IL-27 mAb significantly blunted TAC-induced left-sided pathological remodeling, evidenced by dramatically reduced LV and LA weight-to-tibial length ratios compared to IgG-treated controls (Fig. 7C–Table S6). Furthermore, systemic neutralization of IL-27 effectively mitigated the downstream physical sequelae of left-sided failure, completely suppressing secondary pulmonary congestion (lung weight) and subsequent right-sided compensatory hypertrophy (RV and RA weights) (Fig. 7C–Table S6). Collectively, these preclinical proof-of-concept data establish that targeted pharmacological inhibition of IL-27 is a highly effective therapeutic strategy for halting the structural and functional progression of pressure overload-induced heart failure.

### 3.11 Pharmacological blockade of IL-27 suppresses pathogenic cardiac extracellular matrix remodeling, inflammation, and oxidative stress *in vivo*

To determine whether the macroscopic preservation of cardiac geometry achieved by IL-27 neutralization was matched by a corresponding protection at the microenvironmental level, we evaluated tissue-level remodeling in our pharmacologically treated cohorts. Consistent with the transcriptomic and genetic knockout data, therapeutic intervention with the IL-27 mAb profoundly suppressed adverse extracellular matrix expansion. Sirius Red staining of the left ventricular myocardium at 4 weeks post-TAC revealed extensive interstitial fibrosis in the IgG-treated control mice; however, this pathological collagen deposition was dramatically and significantly attenuated in mice receiving the IL-27 neutralizing antibody (Fig. 8A and B).

**Fig. 8:**
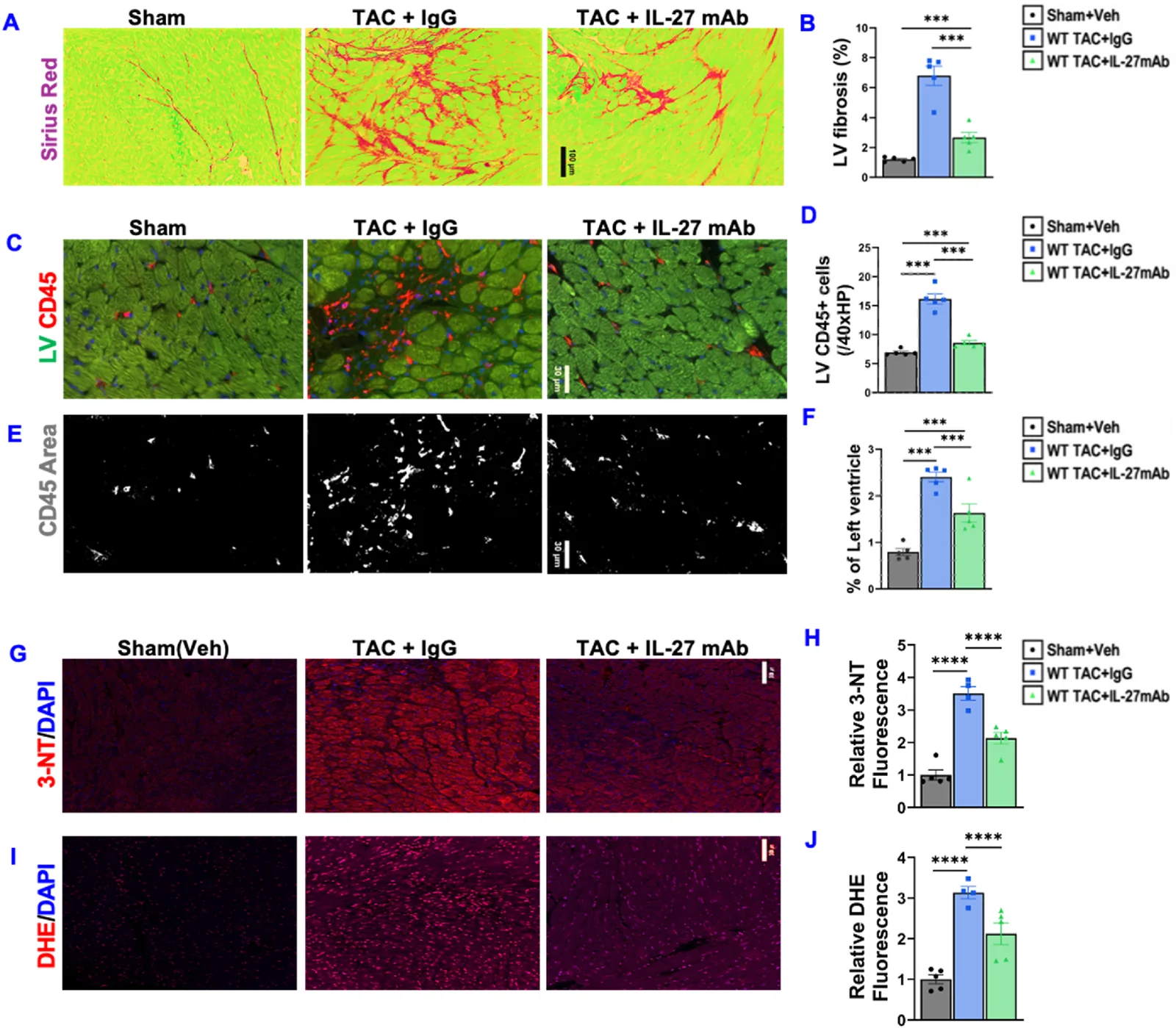
**Pharmacological neutralization of IL-27 mitigates TAC-induced myocardial fibrosis, leukocyte infiltration, and oxidative stress. (A, B)** Representative Sirius Red-stained left ventricular (LV) sections **(A)** and quantification **(B)** demonstrating significantly attenuated interstitial fibrosis in the IL-27 mAb-treated group (TAC + IL27mAb) compared to IgG controls (TAC + IgG) at 4 weeks post-TAC**. (C–F)** Representative immunofluorescence images **(C)**, automated thresholding area maps **(E)**, and corresponding quantification **(D, F)** of CD45^+^ leukocytes, confirming a robust suppression of inflammatory cell infiltration in the TAC + IL27mAb cohort. **(G–J)** Representative images and quantification of LV 3-nitrotyrosine (3-NT) **(G, H)** and dihydroethidium (DHE) **(I, J)** staining. Pharmacological neutralization of IL-27 effectively blunted TAC-induced nitrosative and oxidative stress compared to TAC + IgG controls. (B,D,F,H,J) n= 4-5 per group. Data are presented as mean ± SEM. Statistical significance is denoted by ***p < 0.001, and ****p < 0.0001.

Given the role of the IL-27/IL-27Rα axis in driving myocardial immune responses, we next evaluated leukocyte recruitment and its downstream physiological consequences. Immunofluorescence mapping demonstrated that TAC-induced myocardial inflammation—quantified by both the discrete number of CD45^+^ infiltrating leukocytes (Fig. 8C and D) and the total CD45^+^ proportional area (Fig. 8E and F)—was robustly blunted in the IL-27 mAb cohort compared to the TAC + IgG controls.

Because severe leukocyte infiltration heavily orchestrates the localized oxidative stress cascades that directly impair cardiomyocyte bioenergetics and contractility, we subsequently assessed markers of oxidative and nitrosative damage. In perfect alignment with the mitigated inflammatory burden, therapeutic neutralization of IL-27 significantly suppressed the accumulation of 3-nitrotyrosine (3-NT) (Fig. 8G and H) and blunted reactive oxygen species (ROS) production, as measured by DHE fluorescence (Fig. 8I and J). Together, these histological and immunofluorescent data confirm that pharmacological neutralization of IL-27 successfully halts the interconnected pathophysiological triad of myocardial inflammation, oxidative stress, and interstitial fibrosis during progressive pressure overload.

### 3.12 IL-27 neutralizing antibody suppresses TAC-induced secondary lung immune cell profile

Chronic pressure overload induces significant cardiopulmonary remodeling characterized by systemic and pulmonary immune cell infiltration. To evaluate the impact of therapeutic blockade of IL-27 on pulmonary immune subsets alterations, flow cytometric profiling was performed in lung tissue collected from mice treated with an IL-27 neutralizing monoclonal antibody (IL-27 mAb) or an IgG control. Flow cytometric profiling of lung tissue digests revealed that TAC with IgG administration provoked a marked expansion in pulmonary macrophage accumulation and alterations across multiple leukocyte compartments compared to Sham controls (Supplemental Fig. S7A). TAC did not significantly affect the relation proportions of alveolar macrophages, interstitial macrophages, and monocyte derived macrophages inside macrophages (Supplemental Fig. S8A–D), but caused a significant increase in the proportions of interstitial macrophages and monocyte derived macrophages within the pulmonary CD45^+^ cells (Supplemental Fig. S8E–H). Administration of the IL-27 neutralizing antibody effectively attenuated TAC-driven macrophage accumulation (Supplemental Fig. S7A) and normalized key immune subset distributions toward baseline levels (Supplemental Figs. S7 and S8), demonstrating that IL-27 signaling plays a mechanistic role in coordinating pressure overload-induced pulmonary inflammatory cascades.

### 3.13 IL-27 neutralization attenuates TAC-induced myeloid cell and T cell activation

To investigate whether IL-27 signaling contributes to pressure overload-induced inflammation, mice subjected to TAC were treated with an IL-27 neutralizing antibody (TAC+IL‐27mAb) or control IgG. Flow cytometric analysis revealed that TAC surgery significantly increased the proportion and total number of ${\text{MHCII}}^{\text{high}}$ cells across multiple myeloid compartments, including alveolar macrophages, interstitial macrophages, monocyte-derived macrophages, and ${\text{CD}11c}^{+}$ dendritic cells (Online Supplemental Fig. S9A–L). Administration of IL‐27mAb markedly attenuated these increases, demonstrating that targeted inhibition of the IL-27 axis suppresses antigen-presenting cell activation during pathological cardiac remodeling (Online Supplemental Fig. S9A–L).

To determine the impact of IL-27 signaling on adaptive immune responses during pressure overload, mice subjected to TAC were treated with an IL-27 neutralizing antibody or control IgG. Flow cytometric analysis demonstrated that TAC surgery significantly increased the frequency of activated CD44^high^ cells and decreased CD44^low^ cells across CD3^+^, CD4^+^, and CD8^+^ T cell subsets ( Online Supplemental Fig. S10A–I). Administration of IL-27 mAb effectively attenuated these changes, reducing CD44^high^ proportions and restoring CD44low populations toward baseline levels (Online Supplemental Fig. S10).

### 3.14 Exogenous IL-27 accelerates LV dysfunction and exacerbates pathological remodeling

To definitively confirm the pathogenic role of the IL-27 signaling axis during hemodynamic stress, we employed a complementary gain-of-function approach. Following TAC surgery, wild-type mice were treated with either daily intraperitoneal injections of recombinant mouse IL-27 (rmIL-27; 2.5 μg/mouse) or a saline vehicle control over a 2-week period (Fig. 9A). Pulsed-wave Doppler measurements show that TAC resulted in comparable degrees of aortic constriction between saline-treated and rmIL-27-treated mice (Supplemental Fig. S11).

**Fig. 9:**
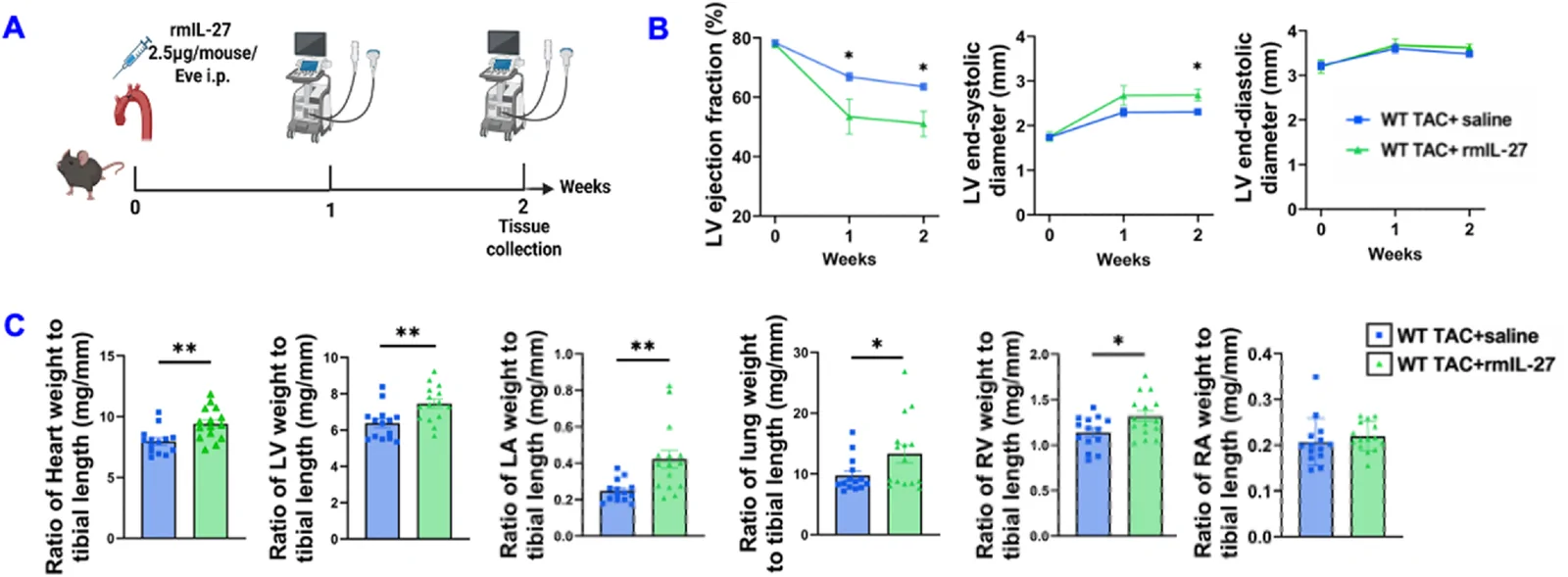
**Exogenous IL-27 administration exacerbates TAC-induced cardiac dysfunction and pathological hypertrophy. (A)** Experimental schematic for the daily intraperitoneal administration of recombinant mouse IL-27 (rmIL-27; 2.5 μg/mouse) or saline vehicle in wild-type mice for 2 weeks following transverse aortic constriction (TAC). **(B)** Longitudinal echocardiographic quantification of left ventricular ejection fraction (LVEF), LV end-systolic diameter, and LV end-diastolic diameter. Treatment with rmIL-27 significantly accelerated the decline in LVEF and exacerbated LV systolic dilation post-TAC compared to saline controls. **(C)** Morphometric quantification of total heart, LV, left atrial (LA), lung, right ventricular (RV), and right atrial (RA) weights normalized to tibial length (mg/mm) at 2 weeks post-TAC. Exogenous rmIL-27 significantly worsened left-sided cardiac hypertrophy, secondary pulmonary congestion, and compensatory RV remodeling. *n = 14-15 per group*. Data are presented as mean ± SEM. *p < 0.05 and **p < 0.01.

Longitudinal echocardiographic assessment revealed that systemic IL-27 supplementation drastically accelerated the progression of cardiac dysfunction. By 2 weeks post-TAC, mice receiving rmIL-27 exhibited a significantly exacerbated decline in left ventricular ejection fraction and a more pronounced pathological increase in LV end-systolic diameter compared to the saline-treated TAC cohort (Fig. 9B; Table S9).

Consistent with this rapid functional deterioration, post-mortem gravimetric analysis demonstrated that exogenous IL-27 severely aggravated macroscopic structural remodeling. Mice treated with rmIL-27 showed significantly elevated total heart, LV, and LA weight-to-tibial length ratios, indicating worsened left-sided multi-chamber hypertrophy (Fig. 9C–Table S8). Furthermore, this accelerated left-sided failure translated into significantly exacerbated downstream sequelae, marked by intensified pulmonary congestion (lung weight) and secondary right ventricular hypertrophy relative to saline controls (Fig. 9C–Table S8). Collectively, these gain-of-function data strongly complement our genetic ablation and pharmacological neutralization studies, unequivocally establishing elevated IL-27 as a critical, direct driver of progressive heart failure and adverse structural remodeling.

### 3.15 Exogenous IL-27 aggravates LV fibrosis, inflammation, and oxidative stress

To determine whether the accelerated functional decline and structural hypertrophy caused by rmIL-27 were driven by exacerbations at the microenvironmental level, we evaluated tissue remodeling, leukocyte recruitment, and oxidative damage in the gain-of-function cohorts. Consistent with our loss-of-function findings, histological evaluation using Sirius Red staining demonstrated that daily administration of rmIL-27 significantly worsened interstitial collagen deposition in the left ventricle at 2 weeks post-TAC compared to saline-treated TAC controls (Fig. 10A and B).

**Fig. 10:**
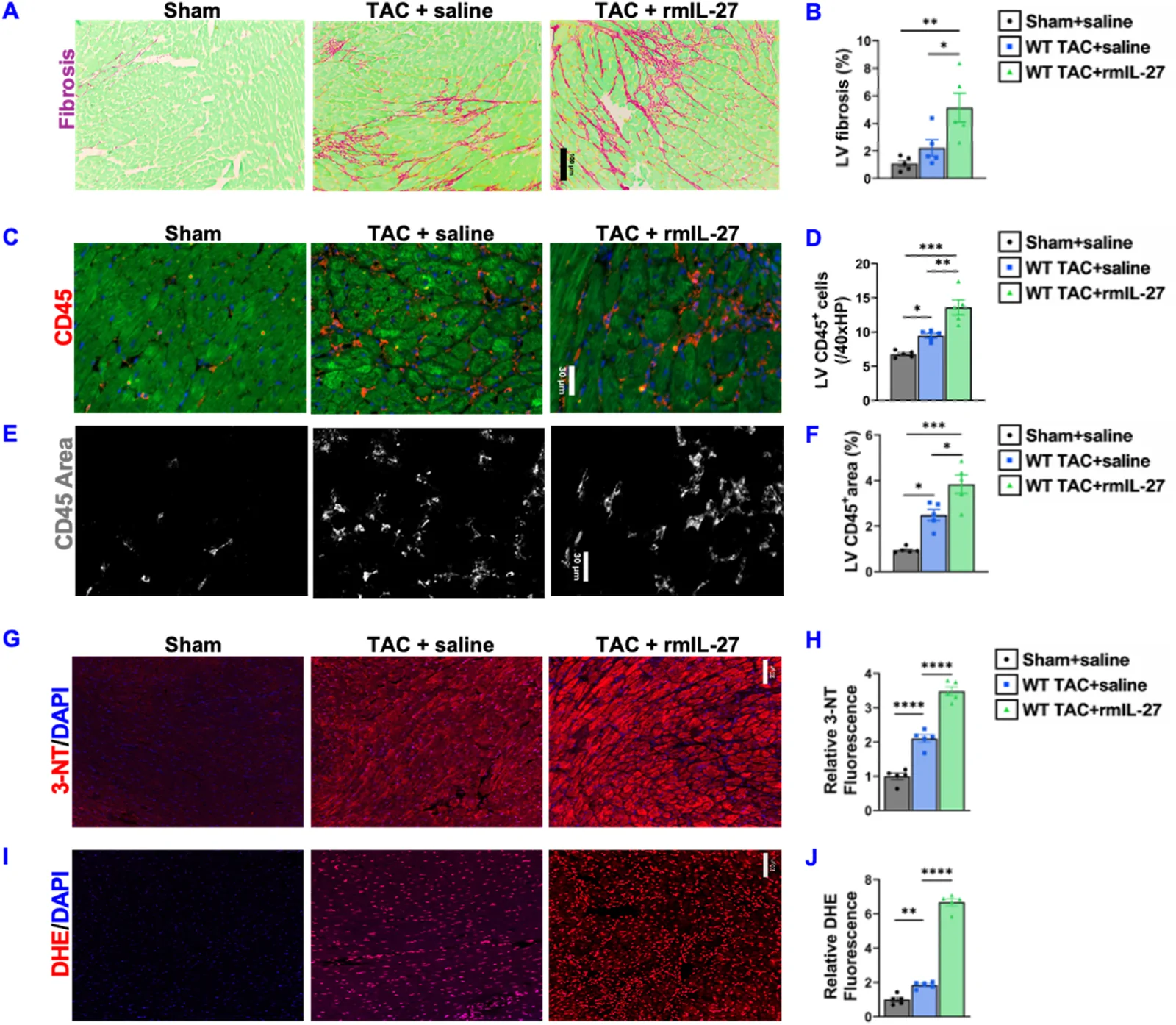
**Exogenous IL-27 administration exacerbates myocardial fibrosis, leukocyte infiltration, and oxidative stress following TAC. (A, B)** Representative Sirius Red-stained left ventricular (LV) sections **(A)** and quantification **(B)** demonstrating that recombinant mouse IL-27 treatment (WT TAC + rmIL27) significantly worsens interstitial collagen deposition compared to saline-treated TAC controls at 2 weeks post-TAC. **(C–F)** Representative immunofluorescence images **(C)**, thresholding area maps **(E)**, and quantification **(D, F)** of CD45^+^ leukocytes, confirming a robust increase in inflammatory cell infiltration with rmIL-27 treatment. **(G–J)** Representative images and quantification of LV 3-nitrotyrosine (3-NT) **(G, H)** and dihydroethidium (DHE) **(I, J)** staining. Exogenous rmIL-27 treatment markedly amplified TAC-induced nitrosative and oxidative stress. n = 5 per group. Data are presented as mean ± SEM. Statistical significance is denoted by * p < 0.05, **p < 0.01, ***p < 0.001, and ****p < 0.0001; ns = not significant.

Furthermore, immunofluorescence mapping revealed that systemic IL-27 supplementation markedly intensified the cardiac inflammatory response. The recruitment of CD45^+^ leukocytes—quantified by both discrete cell counts per high-power field (Fig. 10C and D) and total proportional CD45^+^ area (Fig. 10E and F)—was robustly increased following rmIL-27 treatment.

Because heightened inflammation drives oxidative damage during hemodynamic stress, we subsequently examined markers of nitrosative and oxidative burden. In alignment with their worsened functional status and enhanced fibrosis, mice treated with rmIL-27 exhibited a striking, statistically significant amplification in 3-nitrotyrosine (3-NT) accumulation (Fig. 10G, H) and a sharp elevation in dihydroethidium (DHE) oxidation (Fig. 10I and J). Together, these comprehensive gain-of-function histological and immunofluorescent data confirm that elevated levels of IL-27 actively promote adverse extracellular matrix remodeling, inflammatory cell infiltration, and oxidative stress, solidifying the IL-27 axis as a core pathogenic driver in pressure overload-induced heart failure.

### 3.16 Exogenous IL-27 administration promotes cardiac T cell activation

To gain deeper insight into how systemic IL-27 supplementation modulates the myocardial immune landscape during pressure overload, we performed comprehensive flow cytometric profiling of cardiac immune cell subsets in mice after TAC and under sham conditions. Flow cytometric analysis revealed that the relative proportional distribution of distinct cardiac immune cell subsets remained largely comparable across the groups (Fig. 11A). Given that total cardiac leukocytes are markedly increased following rmIL-27 treatment post-TAC (Fig. 10C and D), the overall number of each individual immune subset—including macrophages, neutrophils, and lymphocytes—is elevated compared to the saline-treated TAC group as illustrated in Fig. 11B.

**Fig. 11:**
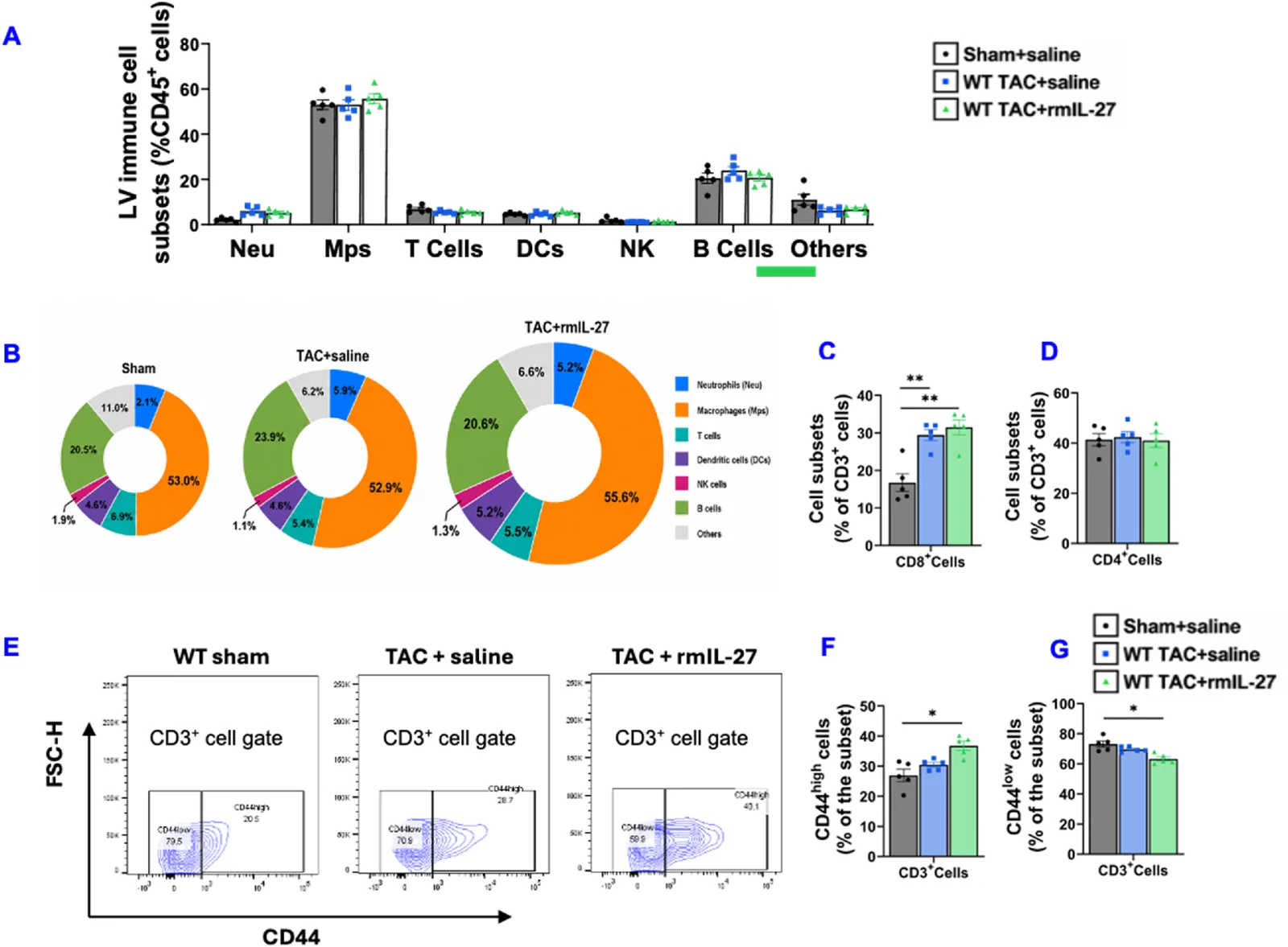
**Exogenous IL-27 administration alters cardiac immune cell composition and increases T cell activation following TAC. (A)** Flow cytometric quantification of left ventricular (LV) immune cell subsets (neutrophils, macrophages, T cells, B cells, dendritic cells, natural killer cells, and others) across Sham, TAC + saline, and TAC + rmIL27 groups. **(B)** Representative conceptual donut charts illustrating that the absolute numbers of each immune subset expand in tandem with increased total leukocytes according to the unchanged percentage of each subset. **(C, D)** Quantitative bar graphs showing the percentages of CD8^+^**(C)** and CD4^+^**(D)** cells as a fraction of CD3^+^ T cells. **(E)** Representative flow cytometry gating plots for CD3^+^ cells showing CD44^low^ and CD44^high^ populations across experimental groups. **(F, G)** Quantitative bar graphs of CD44^high **(F)** and CD44^low^**(G)** cell subsets within the CD3^+^ T cell population, demonstrating a significant increase in activated (CD44^high^) T cells following rmIL-27 administration post-TAC. n = 5/group. Data are presented as mean ± SEM. Statistical significance is denoted by * p < 0.05 and **p < 0.01; ns = not significant.

To further dissect the adaptive immune response, we evaluated T cell subsets within the myocardium. Although the proportions of CD8^+^ and CD4^+^ T cells relative to total CD3^+^ cells showed modest or comparable changes in TAC groups (Fig. 11C and D), evaluation of activation markers revealed a significant shift in CD3^+^ T cell subset in mice treated with IL-27. Specifically, rmIL-27 administration following TAC provoked a significant increase in the frequency of activated CD44^high^ T cells, accompanied by a corresponding reduction in the naïve CD44^low^ T cell fraction within the CD3^+^ population (Fig. 11E–G). Together, these findings indicate that exogenous IL-27 not only amplifies total cardiac leukocyte recruitment during pressure overload but also actively drives the polarization and activation of myocardial T cell responses.

### 3.17 Exogenous IL-27 aggravates TAC-induced pulmonary leukocyte accumulation

Given the prominent role of immune cell infiltration in heart failure-associated lung injury and fibrosis, immunofluorescence analysis of lung tissue was performed to assess overall immune cell accumulation. While TAC induced a modest increase in CD45^+^ immune cells compared to sham controls, systemic treatment with recombinant murine IL-27 (rmIL-27) drastically exacerbated this response (Supplemental Fig. S12). Quantitative evaluation confirmed a marked, statistically significant increase in the density of CD45^+^ cells in the lungs of rmIL-27-treated mice compared to the saline-treated TAC group. These findings indicate that IL-27 strongly promotes pulmonary immune cell recruitment in this model of cardiac pressure overload (Supplemental Fig. S12). The detailed flow cytometry gating strategies used for the identification of cardiac and pulmonary immune cell subsets are illustrated in Supplemental Figs. S13 and S14.

## 4 Discussion

The current study provides comprehensive evidence that IL-27/IL-27R signaling acts as a critical pathogenic driver of pressure overload–induced cardiac inflammation and HF development. The major new findings include: (i) IL-27p28 expression was significantly increased in myocardial samples from heart failure mice; (ii) genetic inhibition of IL-27/IL-27R signaling in IL-27Rα KO mice significantly reduced TAC-induced mortality and mitigated LV inflammation, fibrosis, and dysfunction in both sexes; (iii) pharmacological inhibition via an IL-27 neutralizing antibody significantly attenuated TAC-induced LV inflammation, fibrosis, and dysfunction; (iv) enhancing this signaling with rmIL-27 significantly exacerbated these TAC-induced pathological responses; (v) IL-27Rα KO and IL-27 neutralization effectively abolished TAC-induced LV oxidative stress, whereas administration of rmIL-27 promoted it; (vi) bulk RNA sequencing and network-based bioinformatics of LV tissues demonstrated that genetic deletion protected hearts from TAC-induced transcriptomic alterations, specifically downregulating key driver networks associated with innate immune activation, cytokine signaling, and extracellular matrix remodeling; and (vii) IL-27Rα KO and IL-27 neutralizing antibody treatment successfully attenuated TAC-induced increases in lung weight and pulmonary inflammation. Together, these data provide solid evidence that IL-27/IL-27R signaling acts as an important upstream driver orchestrating systolic overload-induced sterile cardiac inflammation, oxidative damage, and subsequent ventricular and pulmonary dysfunction.

Our findings demonstrate that both genetic and pharmacological inhibition of the IL-27/IL-27R signaling pathway simultaneously attenuated cardiac oxidative stress and inflammation. Conversely, treating mice with recombinant mouse IL-27 exacerbated both cardiac oxidative stress and inflammatory responses. These results indicate a central role for the IL-27 axis in *promoting* the pathological milieu during pressure overload-induced remodeling, *although TAC also caused increases of a few proinflammatory genes in IL-27Ra KO mice after TAC*. *Given IL-27Ra broadly expressed multiple immune and nonimmune cells in various tissues, the observed cardiac protective effect in the IL-27R KO mice after TAC is likely a collective effect on multiple systems in these mice.*

It is well established that oxidative stress plays a fundamental role in triggering cardiac injury and the progression to heart failure [42], [43], [44]. Oxidative stress and inflammation operate in a destructive, self-amplifying feedforward loop that accelerates myocardial injury and drives the progression to heart failure. This excessive generation of reactive oxygen species and concomitant inflammation operate in a destructive, self-amplifying feedforward loop that accelerates myocardial injury [45]. Conversely, enhancing defenses against oxidative stress (such as through antioxidant M40401, a high-selenium diet, enhanced antioxidant expression, and iNOS inhibition) successfully attenuates pressure overload-induced cardiopulmonary oxidative stress, inflammation, and heart failure [24], [44], [46], [47], [48]. Specifically, excess ROS induce direct damage to cardiac myocytes and endothelial cells, triggering the release of damage-associated molecular patterns (DAMPs) that stimulate the innate immune system [49]. Furthermore, elevated oxidative stress enhances chronic tissue damage by releasing sequestered endogenous antigens and chemically modifying self-peptides into neoantigens, thereby driving adaptive autoimmune-like responses and sustaining chronic inflammation [50]. Consistent with the notion that neoantigens promote systolic overload-induced cardiac failure, our previous works shows that CD8^+^ T cells, antigen presenting cells, and T cell action exert important roles in promoting cardiac inflammation, HF development and HF progression [18], [20], [21].

Conversely, activated innate and acquired immune mechanisms actively drive and amplify cellular oxidative stress and myocardial injury. Infiltrating innate immune cells, such as activated macrophages and monocytes, directly release large amounts of ROS via an oxidative burst. This massive ROS production is heavily driven by mechanisms including NADPH oxidase activation and iNOS uncoupling, which subsequently precipitate the formation of highly toxic oxidants like peroxynitrite and actively contribute to iNOS-induced heart failure [51]. Furthermore, increased oxidative stress has been shown to directly upregulate PDE5 expression to reduced cGMP/PKG signaling in the failing heart [52]. Chronic activation of these enzymatic pathways severely exacerbates maladaptive remodeling, whereas targeted inhibition of PDE5 can prevent and reverse cardiac hypertrophy [53]. Simultaneously, acquired immune components recruit additional inflammatory cells to the myocardium, upregulate tissue-damaging oxidases, and secrete profibrotic mediators that further heighten intracellular ROS generation. Indeed, our previous studies showed that inhibition of cardiac inflammation by either genetic or pharmacological inhibition of IL-12β significantly attenuated HF development and/or cardiac oxidative stress [24], [46]. Therefore, IL-27 signaling likely promotes cardiac injury by exacerbating these interconnected factors, and its targeted inhibition protects the myocardium by reducing both oxidative stress and inflammation.

A striking aspect of our study is the pronounced pro-inflammatory phenotype of IL-27 in the failing heart, which contrasts with its well-characterized immunoregulatory functions in autoimmunity and infection—such as suppressing Th17 responses or inducing IL-10 [28], [34]. This discrepancy emphasizes the highly context-dependent nature of cytokine signaling in modulating inflammatory responses [28], [34]. Consistent with this context dependence, while IL-27R signaling has been reported to limit atherosclerosis in Ldlr^−/−^ mice [54], our findings indicate that it plays a pathogenic role in promoting cardiac injury during pressure overload-induced HF. Given that IL-27R is highly abundant across myeloid and lymphoid populations, these data suggest that within the specific biomechanical and sterile inflammatory environment of cardiac pressure overload, IL-27/IL-27R signaling may act predominantly to enhance the pro-inflammatory and tissue-damaging capacity of infiltrating immune cells, thereby likely driving maladaptive myocardial remodeling.

Beyond immune modulation, our transcriptomic analysis, reinforced by GSEA and KDA, reveals a profound role for IL-27 in promoting cardiac fibrosis in response to systolic pressure overload. While exogenous IL-27 enhanced fibrotic deposition, targeted inhibition or genetic ablation of IL-27 signaling consistently attenuated cardiac fibrosis and normalized ECM gene programs (e.g., *Col1a1*, *Postn*), a pathological process often modulated by *TGF-β* signaling pathway [55], [56]. Mechanistically, we hypothesize a dual-pronged profibrotic effect. First, IL-27 may directly stimulate fibrocyte and resident fibroblast activation [57], [58]. This direct axis is supported by evidence across different tissues and disease models; for example, IL-27 significantly increases proliferation and type I collagen synthesis in dermal fibroblasts from systemic sclerosis patients [59] and activates JAK/STAT signaling in cardiac fibroblasts to promote ECM production following myocardial infarction [60]. Second, we speculate that IL-27 promotes extracellular remodeling indirectly by enhancing local tissue inflammation and recruiting profibrotic macrophages and T cells [13], [61]. While current antifibrotic strategies often focus on the classical TGF-β signaling pathway with highly variable clinical outcomes [62], our data identify IL-27 as a potent, inflammation-driven profibrotic signal that may function distinctly from, or synergistically with, these classical pathways. Ultimately, the robust regulation of the cardiac ECM by IL-27 implies a critical immune-fibrotic crosstalk that warrants future cell-specific mechanistic investigation [57], [58].

Importantly, our findings highlight the efficacy of targeted IL-27 blockade in mitigating the pathophysiological transition from LV failure to pulmonary remodeling and right ventricular hypertrophy. Pulmonary vascular remodeling remains a hallmark of advanced HF and serves as a primary determinant of mortality [5], [6], [63]. In our model, IL-27 inhibition significantly attenuated pulmonary vascular muscularization and localized tissue inflammation. The mechanisms underlying this robust protection are likely twofold: an indirect hemodynamic benefit derived from preserved LV function—thereby attenuating retrograde pulmonary venous pressure—coupled with a direct anti-inflammatory effect within the pulmonary microenvironment. This direct effect is evidenced by a markedly reduced antigen-presenting capacity, indicated by attenuated MHC-II expression, among pulmonary macrophages. Consequently, these data position IL-27 inhibition as a comprehensive therapeutic strategy that not only protects the ventricular myocardium but also preserves the structural integrity of the pulmonary vascular bed, potentially delaying the onset of heart failure-induced pulmonary remodeling.

It is noteworthy that the cardioprotective effects of the IL-27 neutralizing antibody on functional parameters were less profound than those observed in genetic knockout model. We postulate that this discrepancy arises from differing degrees of pathway inhibition. While genetic ablation completely abolishes the IL-27/IL-27R signaling axis, pharmacological intervention with a neutralizing antibody likely achieves only a partial or incomplete blockade within the overloaded myocardium. This incomplete inhibition appears sufficient to significantly attenuate histological markers of adverse remodeling but may not be robust enough to fully rescue cardiac function to the extent seen with total genetic deletion.

While our study provides strong evidence that IL-27/IL-27R signaling acts as a major pathogenic driver of pressure overload–induced cardiac failure, several limitations warrant consideration. First, because IL-27 signaling promotes TAC-induced cardiac inflammation, oxidative stress, and fibrosis—each capable of independently driving HF progression—our experimental design precludes an exact determination of their relative, independent contributions. Second, although left ventricular failure often progresses to pulmonary inflammation and RV hypertrophy, localized lung inflammation can also directly drive these processes independent of LV dysfunction [5], [64], [65]. Thus, we cannot definitively determine whether the observed reduction in pulmonary remodeling is partially mediated by a direct attenuation of primary lung inflammation. Third, to induce comparable disease severity, we utilized a 26-gauge needle for male mice and a smaller 27-gauge needle for female mice, as females are generally more resistant to TAC-induced HF [57], [58]. Furthermore, the inherent differences in baseline body weight and growth trajectories between young adult male and female mice complicate direct sex comparisons; even if identical needle sizes were utilized, the imposed hemodynamic stress would disproportionately scale with body mass, unavoidably confounding the assessment of sex-specific responses [24]. Consequently, while this precludes a direct evaluation of sex-dependent differences, our findings unequivocally demonstrate that IL-27Rα deletion confers robust protection in both sexes. Finally, clinical heart failure typically arises from a complex interplay of multiple, chronic cardiovascular risk factors in aging populations; therefore, our reliance on a singular mechanical pressure overload model in young, healthy mice may not fully recapitulate the multifaceted etiology of human clinical disease.

In summary, we have identified IL-27 as a critical, maladaptive checkpoint in the systolic overload-induced heart failure. Thus, targeting this specific pathway offers a viable clinical avenue to suppress pathological inflammation, fibrosis, and dysfunction in the setting of pressure overload.

## CRediT authorship contribution statement

**Ziru Niu:** Writing – original draft, Validation, Methodology, Investigation, Formal analysis, Data curation. **Umesh Bhattarai:** Investigation, Formal analysis, Data curation. **Dongzhi Wang:** Investigation. **Xiaochen He:** Methodology, Investigation. **Lihong Pan:** Investigation. **Heng Zeng:** Writing – review & editing, Methodology. **Jian-Xiong Chen:** Writing – review & editing. **John S. Clemmer:** Writing – review & editing. **Lei Hou:** Writing – review & editing, Writing – original draft. **Yingjie Chen:** Writing – review & editing, Supervision, Project administration, Conceptualization.

## Ethics declaration

This study was conducted in accordance with the ARRIVE (Animal Research: Reporting of In Vivo Experiments) guidelines. This study was approved by the University of Mississippi Medical Center. (Approval No. 2154)

## Declaration of competing interest

The authors declare that they have no known competing financial interests or personal relationships that could have appeared to influence the work reported in this paper.

## Data Availability

Data will be made available on request.
