# Supplementary material for: IL-27 promotes systolic overload-induced cardiac oxidative stress, inflammation, fibrosis, and heart failure development
Source: Redox Biol. 2026 Oct 28;98:104459. doi: 10.1016/j.redox.2026.104459 (PMC13760151; doi:10.1016/j.redox.2026.104459)
Supplement: Supplementary file 1 [file mmc1.pdf]

IL-27 promotes systolic overload-induced cardiac oxidative stress, inflammation, fibrosis, and heart failure development

[1,2#]Ziru Niu, [2,3#]Umesh Bhattarai, [2#]Dongzhi Wang, [1]Xiaochen He, [1]Lihong Pan, [4]Heng Zeng, [4]Jian-Xiong Chen, [2]John S. Clemmer, [1]Lei Hou[*], [2]Yingjie Chen[*]

[1] Department of Cardiology, Songjiang Hospital Affiliated to Shanghai Jiao Tong University School of Medicine, Shanghai, China

[2] Department of Physiology and Biophysics, School of Medicine, University of Mississippi Medical Center, Jackson, MS, United States

[3] Division of Clinical Pharmacology, Department of Medicine, Indiana University School of Medicine, Indianapolis, Indiana, USA

[4] Department of Pharmacology and Toxicology, School of Medicine, University of Mississippi Medical Center, Jackson, MS, United States

Total word count:

Running Title: IL-27 and pressure overload-induced cardiac failure

Conflict of Interest: None.

[#] These authors contributed equally to this work.

***Correspondence:**

Yingjie Chen, Ph.D. Email: ychen2@umc.edu

Lei Hou, MD, Ph.D. Email: Dr_houlei@shsmu.edu.cn

Supplementary Table 1: Antibodies used for flow cytometry analyses

Antibody	Conjugate	Clone	Vendor	Catalog #
**CD3e**	BUV737	145-2C11	BD Biosciences	612771
**CD4**	BUV496	GK1.5	BD Biosciences	612952
**CD8α**	BB790	53-6.7	BD Biosciences	624296
**CD11b**	BV650	M1/70	Biolegend	101259
**CD11c**	BV711	N418	Biolegend	117349
**CD16/32**	-	93	Biolegend	101302
**CD19**	BUV395	1D3	BD Biosciences	563557
**CD44**	FITC	IM-7	BD Biosciences	553133
**CD45**	BUV805	30-F11	BD Biosciences	568336
**CD62L**	AF700	MEL-14	BD Biosciences	104418
**F4/80**	BUV563	T45-2342	BD Biosciences	749284
**I-A/I-E (MHC-II)**	APC-Cy7	M5/114.15.2	Biolegend	107628
**Ly6C**	BV605	AL-21	BD Biosciences	563011
**γδ T-Cell Receptor**	BUV661	GL3	BD Biosciences	750410
**Ly6G**	AF700	1A8	Biolegend	127622
**CXCR6**	BV711	SA051D1	BioLegend	151111
**NK1.1**	APC	PK136	eBioscience	17-5941-82

Table 2. Effect of IL-27 receptor deficiency on TAC-induced increases of tissue weight, and their ratios to bodyweight or tibial length in male mice.

Parameters	Male WT Sham (n=10)	Male IL-27R KO Sham (n=10)	Male WT TAC (n=10)	Male IL-27R KO TAC (n=19)
Body Weight (g)	30.89±0.54	30.09±1.02	31.61±0.65	30.07±0.55
Tibial Length (mm)	18.21±0.11	17.96±0.09	18.42±0.06	17.98±0.07 #
LV Weight (mg)	111.1±1.94	97.80±3.14	198.0±9.62 *	135.4±6.21 *#
LA Weight (mg)	4.09±0.32	3.01±0.30	12.43±1.88 *	6.04±1.28 #
Lung Weight (mg)	163.7±16.52	159.3±11.72	290.8±34.78 *	168.7±13.00 #
RV Weight (mg)	18.56±0.48	20.60±1.01	30.17±2.57 *	23.26±1.56 #
RA Weight (mg)	3.60±0.31	3.08±0.22	4.64±0.44	3.49±0.23 #
Heart Weight (mg)	137.3±2.35	124.5±4.05	245.2±13.73 *	168.2±8.37 *#
LV Weight/BW (mg/g)	3.60±0.06	3.26±0.08	6.29±0.35 *	4.53±0.22 *#
LA Weight/BW (mg/g)	0.13±0.01	0.10±0.01	0.40±0.07 *	0.21±0.05 #
Lung Weight/BW (mg/g)	5.25±0.42	5.32±0.37	9.32±1.26 *	5.69±0.53 #
RV Weight/BW (mg/g)	0.60±0.01	0.68±0.02	0.97±0.10 *	0.78±0.06
RA Weight/BW (mg/g)	0.12±0.01	0.10±0.01	0.15±0.02	0.12±0.01

Data are presented as mean ± SEM; n represents the number of mice. Data were analyzed by two-way ANOVA, followed by Bonferroni-adjusted multiple-comparisons tests. *P<0.05 versus the corresponding Sham control; #P<0.05 versus WT TAC. All male groups were collected at 6 weeks. TAC, transverse aortic constriction; IL-27R, interleukin-27 receptor; KO, knockout; BW, body weight; LV, left ventricle; LA, left atrium; RV, right ventricle; RA, right atrium.

Table 3. Left ventricular dimension and function in WT and IL-27Ra KO male mice at baseline and 2, 4, and 6 weeks after TAC

Parameters	Before TAC WT	Before TAC IL-27R KO	TAC 2 weeks WT	TAC 2 weeks IL-27R KO	TAC 4 weeks WT	TAC 4 weeks IL-27R KO	TAC 6 weeks WT	TAC 6 weeks IL-27R KO
Heart Rate (bpm)	566±12	574±16	580±10	545±6#	575±11	576±7	552±11	565±12
LVID;s (mm)	2.00±0.09	2.04±0.09	2.95±0.13*	*2.41±0.07*#*	3.12±0.14*	*2.48±0.08*#*	3.41±0.20*	*2.48±0.10*#*
LVID;d (mm)	3.39±0.08	3.53±0.07	4.22±0.10*	*3.83±0.07*#*	4.16±0.10*	*3.75±0.08*#*	4.42±0.15*	3.64±0.09#
LV Volume;s (μL)	13.24±1.58	13.83±1.52	34.65±3.96*	*21.06±1.64*#*	39.66±3.86*	*22.79±1.90*#*	49.98±6.57*	*22.91±2.41*#*
LV Volume;d (μL)	47.54±2.81	52.27±2.54	80.37±4.57*	*63.66±2.70*#*	77.26±4.08*	*60.76±3.26*#*	90.17±7.03*	56.72±3.38#
Ejection Fraction (%)	72.79±1.60	73.99±2.00	57.10±4.14*	*67.35±1.48*#*	49.86±3.05*	*63.31±1.53*#*	46.82±3.43*	*61.08±1.80*#*
Fractional Shortening (%)	41.08±1.32	42.33±1.67	30.25±2.42*	*37.06±1.08*#*	25.30±1.94*	*33.94±1.07*#*	23.57±2.02*	*32.29±1.19*#*
LVPW;s (mm)	1.40±0.05	1.43±0.05	1.31±0.04	1.41±0.03	1.40±0.05	1.36±0.03	1.38±0.06	1.41±0.03
LVPW;d (mm)	0.90±0.06	0.95±0.05	0.88±0.04	0.90±0.03	1.05±0.05	0.93±0.03	1.07±0.04*	1.07±0.04

Data are presented as mean ± SEM; n represents individual animals. Data were analyzed by two-way ANOVA, followed by Bonferroni-adjusted multiple-comparisons tests. *P<0.05 versus the corresponding Before TAC group; #P<0.05 versus time-matched WT TAC. TAC, transverse aortic constriction; IL-27Ra, interleukin-27 receptor; KO, knockout; LVID, left ventricular internal diameter; LV, left ventricle; LVAW, left ventricular anterior wall; LVPW, left ventricular posterior wall; s, systole; d, diastole.

Table 4. Effect of IL-27Ra knockout on TAC-induced increases of tissue weight, and their ratios to bodyweight or tibial length in female mice.

Parameters	Female WT Sham (n=7)	Female IL-27R KO Sham (n=6)	Female WT TAC (n=9)	Female IL-27R KO TAC (n=14)
Body Weight (g)	25.29±0.61	25.48±0.70	22.83±0.63	27.48±0.99 #
Tibial Length (mm)	17.53±0.08	17.62±0.10	17.57±0.09	17.73±0.04
LV Weight (mg)	79.74±2.65	86.85±6.20	133.4±9.49 *	129.7±4.41 *
LA Weight (mg)	2.61±0.14	2.47±0.14	10.54±3.60 *	5.26±0.97
Lung Weight (mg)	144.5±4.66	143.8±6.03	239.7±50.03	218.6±34.11
RV Weight (mg)	15.69±0.62	16.57±0.83	20.79±2.18	19.66±0.91
RA Weight (mg)	2.87±0.08	2.72±0.20	4.37±0.65 *	3.04±0.20 #
Heart Weight (mg)	100.9±2.86	108.6±6.70	169.1±14.59 *	157.6±6.15 *
LV Weight/BW (mg/g)	3.15±0.05	3.43±0.30	5.89±0.49 *	4.81±0.26 *
LA Weight/BW (mg/g)	0.103±0.005	0.097±0.006	0.492±0.194	0.200±0.041
Lung Weight/BW (mg/g)	5.71±0.06	5.66±0.27	10.90±2.59	8.22±1.43
RV Weight/BW (mg/g)	0.62±0.03	0.65±0.04	0.93±0.13 *	0.73±0.05
RA Weight/BW (mg/g)	0.114±0.004	0.106±0.006	0.197±0.036 *	0.113±0.009 #

Data are presented as mean ± SEM; n represents the number of mice. Data were analyzed by two-way ANOVA, followed by Bonferroni-adjusted multiple-comparisons tests. *P<0.05 versus the corresponding Sham control; #P<0.05 versus WT TAC. All female groups were collected at 4 weeks. TAC, transverse aortic constriction; IL-27R, interleukin-27 receptor; KO, knockout; BW, body weight; LV, left ventricle; LA, left atrium; RV, right ventricle; RA, right atrium.

Table 5. Effect of IL-27Ra knockout on left ventricular dimension and function at baseline and 2 and 4 weeks after TAC in female mice

**Parameters**	**WT before TAC**	**IL-27R KO before TAC**	**WT 2W after TAC**	**IL-27R KO 2W after TAC**	**WT 4W after TAC**	**IL-27R KO 4W after TAC**
Heart Rate (bpm)	583±13	551±8	576±10	563±9	605±22	556±10
LVID;s (mm)	1.86±0.06	1.90±0.10	3.05±0.13*	*2.47±0.09*#*	3.30±0.23*	*2.70±0.16*#*
LVID;d (mm)	3.03±0.09	3.24±0.09	3.92±0.09*	3.71±0.06*	4.08±0.19*	3.79±0.11*
LV Volume;s (μL)	10.74±0.95	11.67±1.57	37.33±3.91*	*22.63±2.35*#*	46.98±7.71*	29.69±4.98*
LV Volume;d (μL)	36.43±2.64	42.78±2.73	67.06±3.50*	59.02±2.24*	75.42±8.26*	62.98±4.52*
Ejection Fraction (%)	70.59±1.41	73.41±2.26	45.28±3.63*	*62.73±2.31*#*	40.21±4.47*	*56.15±3.72*#*
Fractional Shortening (%)	38.80±1.13	41.60±1.81	22.45±2.01*	*33.62±1.48*#*	19.65±2.35*	*29.50±2.26*#*
LVPW;s (mm)	1.22±0.04	1.18±0.03	1.25±0.07	*1.47±0.04*#*	1.23±0.04	*1.43±0.04*#*
LVPW;d (mm)	0.84±0.04	0.77±0.02	0.97±0.07	1.02±0.04*	1.01±0.04*	1.07±0.04*

Data are presented as mean ± SEM; n represents individual animals. Data were analyzed by two-way ANOVA, followed by Bonferroni-adjusted multiple-comparisons tests. *P<0.05 versus the corresponding Before TAC group; #P<0.05 versus time-matched WT TAC. TAC, transverse aortic constriction; IL-27Ra, interleukin-27 receptor; KO, knockout; LVID, left ventricular internal diameter; LV, left ventricle; LVAW, left ventricular anterior wall; LVPW, left ventricular posterior wall; s, systole; d, diastole.

Table 6. Effect of pharmacological blockade of IL-27 signaling on TAC-induced increases of tissue weight, and their ratios to bodyweight or tibial length in male mice.

Parameters	WT Male Control (n=6)	WT TAC+IgG (n=13)	WT TAC+anti-IL-27 Ab (n=11)
Body Weight (g)	25.73±0.80	23.01±0.75 *	23.54±0.25
Tibial Length (mm)	16.93±0.09	16.57±0.10 *	16.64±0.05
LV Weight (mg)	87.57±2.73	162.0±4.71 *	119.6±5.68 *#
LA Weight (mg)	3.50±0.22	17.36±3.55 *	5.52±0.69 #
Lung Weight (mg)	146.4±3.25	303.2±38.84 *	140.2±3.07 #
RV Weight (mg)	19.50±0.93	25.68±1.15 *	19.41±0.63 #
RA Weight (mg)	3.10±0.15	4.90±0.38 *	3.27±0.18 #
Total Heart Weight (mg)	113.7±3.00	209.9±8.39 *	147.8±6.55 *#
LV Weight/BW (mg/g)	3.41±0.06	7.13±0.32 *	5.08±0.22 *#
LA Weight/BW (mg/g)	0.14±0.01	0.81±0.19 *	0.23±0.03 #
Lung Weight/BW (mg/g)	5.70±0.12	13.86±2.13 *	5.96±0.11 #
RV Weight/BW (mg/g)	0.76±0.03	1.14±0.08 *	0.83±0.03 #
RA Weight/BW (mg/g)	0.12±0.01	0.22±0.02 *	0.14±0.01 #

Data are presented as mean ± SEM; n represents the number of mice. Data were analyzed by one-way ANOVA followed by Bonferroni-adjusted tests for three prespecified pairwise comparisons. *P<0.05 versus WT Male Control; #P<0.05 versus WT TAC+IgG. TAC, transverse aortic constriction; IgG, immunoglobulin G; IL-27, interleukin-27; Ab, antibody; BW, body weight; LV, left ventricle; LA, left atrium; RV, right ventricle; RA, right atrium.

Table 7. Effect of IL-27 blocking antibody on left ventricular dimension and function at 2 and 4 weeks after TAC in male mice

Parameters	TAC + IgG 2 weeks	TAC + anti-IL-27 Ab 2 weeks	TAC + IgG 4 weeks	TAC + anti-IL-27 Ab 4 weeks
Heart Rate (bpm)	574±9	578±11	557±10	557±8
LVID;s (mm)	2.98±0.16	2.13±0.08*	3.29±0.11	2.69±0.08*
LVID;d (mm)	3.90±0.10	3.37±0.07*	4.21±0.07	3.75±0.07*
LV Volume;s (µL)	36.63±4.41	15.33±1.58*	44.88±3.47	27.16±1.93*
LV Volume;d (µL)	66.80±3.79	46.85±2.40*	79.30±3.07	60.46±2.75*
Ejection Fraction (%)	47.49±4.48	67.66±2.32*	43.76±3.44	55.25±2.26*
Fractional Shortening (%)	24.28±2.73	37.03±1.69*	21.76±1.95	28.36±1.41*
LVPW;s (mm)	1.42±0.04	1.40±0.04	1.42±0.03	1.38±0.04
LVPW;d (mm)	1.07±0.05	0.94±0.04	1.12±0.04	1.01±0.04

Data are presented as mean ± SEM; n represents individual animals. Data were analyzed by one-way ANOVA followed by Bonferroni-adjusted tests for three prespecified pairwise comparisons. *P<0.05 versus the time-matched TAC + IgG group. TAC, transverse aortic constriction; IgG, immunoglobulin G; IL-27, interleukin-27; Ab, antibody; LVID, left ventricular internal diameter; LV, left ventricle; LVAW, left ventricular anterior wall; LVPW, left ventricular posterior wall; s, systole; d, diastole.

Table 8. Effect of recombinant mouse interleukin-27 (rmIL-27) on TAC-induced increases of tissue weight, and their ratios to bodyweight or tibial length in male mice.

**Parameters**	**WT Male Control (n=5)**	**TAC + Saline (n=14)**	**TAC + rmIL-27 (n=15)**
Body Weight (g)	24.08±0.72	23.92±1.04	23.91±0.92
Tibial Length (mm)	16.68±0.08	17.07±0.25	17.07±0.20
LV Weight (mg)	83.76±3.01	108.7±4.16 *	127.5±5.05 *#
LA Weight (mg)	2.72±0.20	4.21±0.27	7.22±0.84 *#
Lung Weight (mg)	137.2±4.23	165.0±11.31	226.5±25.79 #
RV Weight (mg)	20.20±1.12	19.44±0.89	22.56±1.12
RA Weight (mg)	3.40±0.36	3.54±0.24	3.74±0.14
Total Heart Weight (mg)	110.1±4.11	135.9±4.93	161.0±6.63 *#
LV Weight/BW (mg/g)	3.48±0.11	4.62±0.22 *	5.39±0.23 *#
LA Weight/BW (mg/g)	0.11±0.01	0.18±0.01	0.31±0.04 *#
Lung Weight/BW (mg/g)	5.71±0.16	7.16±0.69	9.82±1.26
RV Weight/BW (mg/g)	0.84±0.05	0.82±0.04	0.95±0.05
RA Weight/BW (mg/g)	0.14±0.01	0.15±0.01	0.16±0.01

Data are presented as mean ± SEM; n represents the number of mice. Data were analyzed by one-way ANOVA followed by Bonferroni-adjusted tests for three prespecified pairwise comparisons. *P<0.05 versus WT Male Control; #P<0.05 versus TAC + Saline. TAC, transverse aortic constriction; rmIL-27, recombinant mouse interleukin-27; BW, body weight; LV, left ventricle; LA, left atrium; RV, right ventricle; RA, right atrium.

Table 9. Effect of recombinant mouse interleukin-27 (rmIL-27) on left ventricular dimension and function in male mice at 1 and 2 weeks after TAC

Parameters	TAC + Saline 1 week	TAC + rmIL-27 1 week	TAC + Saline 2 weeks	TAC + rmIL-27 2 weeks
Heart Rate (bpm)	609±9	618±10	609±10	623±11
LVID;s (mm)	2.30±0.10	2.67±0.22	2.31±0.06	2.68±0.13*
LVID;d (mm)	3.60±0.11	3.68±0.14	3.48±0.05	3.62±0.08
LV Volume;s (µL)	18.58±1.97	29.23±7.10	18.63±1.22	28.22±3.45*
LV Volume;d (µL)	55.14±4.03	58.46±6.18	50.54±1.87	55.92±3.03
Ejection Fraction (%)	66.91±1.41	53.46±5.93	63.55±1.22	51.08±4.28*
Fractional Shortening (%)	36.38±1.05	27.98±3.49*	33.79±0.86	26.28±2.51*
LVPW;s (mm)	1.25±0.05	1.30±0.04	1.30±0.04	1.36±0.04
LVPW;d (mm)	0.75±0.04	0.91±0.06*	0.86±0.03	1.02±0.05*

Data are presented as mean ± SEM; n represents individual animals. Data were analyzed by one-way ANOVA followed by Bonferroni-adjusted tests for three prespecified pairwise comparisons. *P<0.05 versus the time-matched TAC + Saline group. TAC, transverse aortic constriction; rmIL-27, recombinant mouse interleukin-27; LVID, left ventricular internal diameter; LV, left ventricle; LVAW, left ventricular anterior wall; LVPW, left ventricular posterior wall; s, systole; d, diastole.

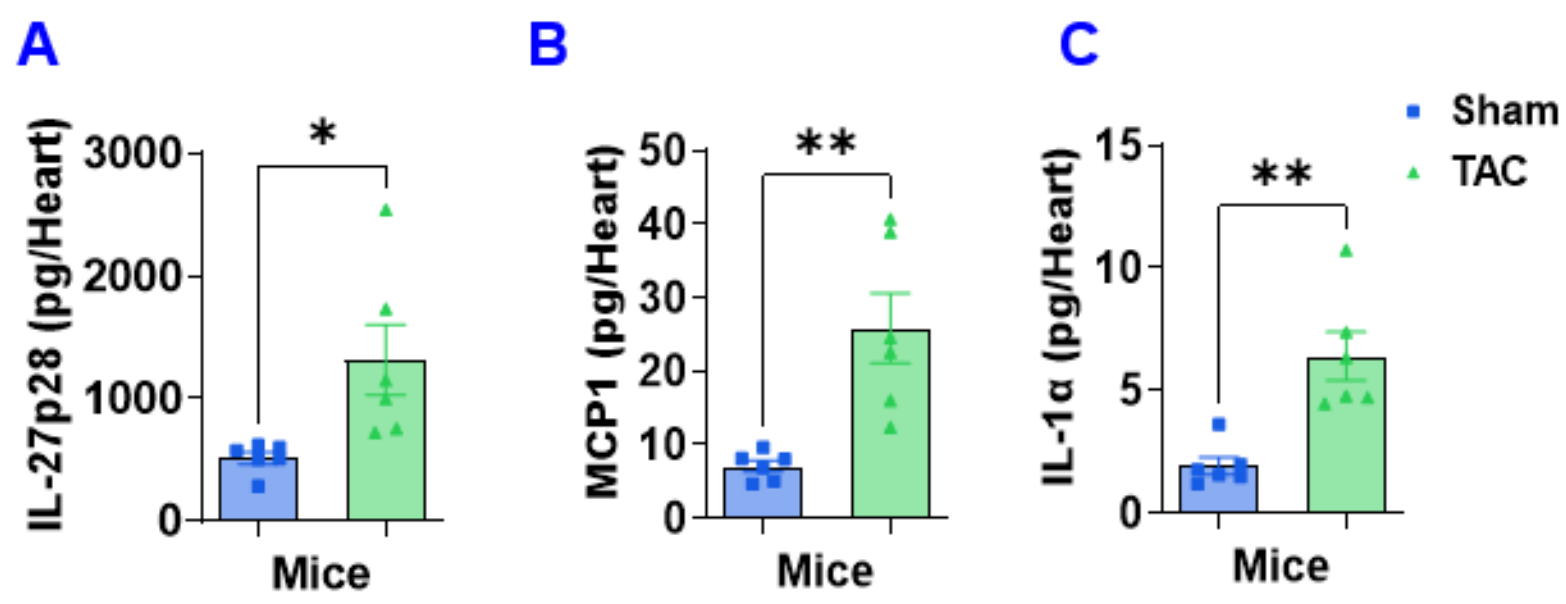

Supplemental Figure 1: IL-27 and inflammatory cytokines are upregulated in murine heart failure. **(A-C)** Quantification of IL-27p28, MCP1, and IL-1α protein levels (pg/heart) in the cardiac tissue of mice following Sham or TAC surgery. *n = 6 per group.*

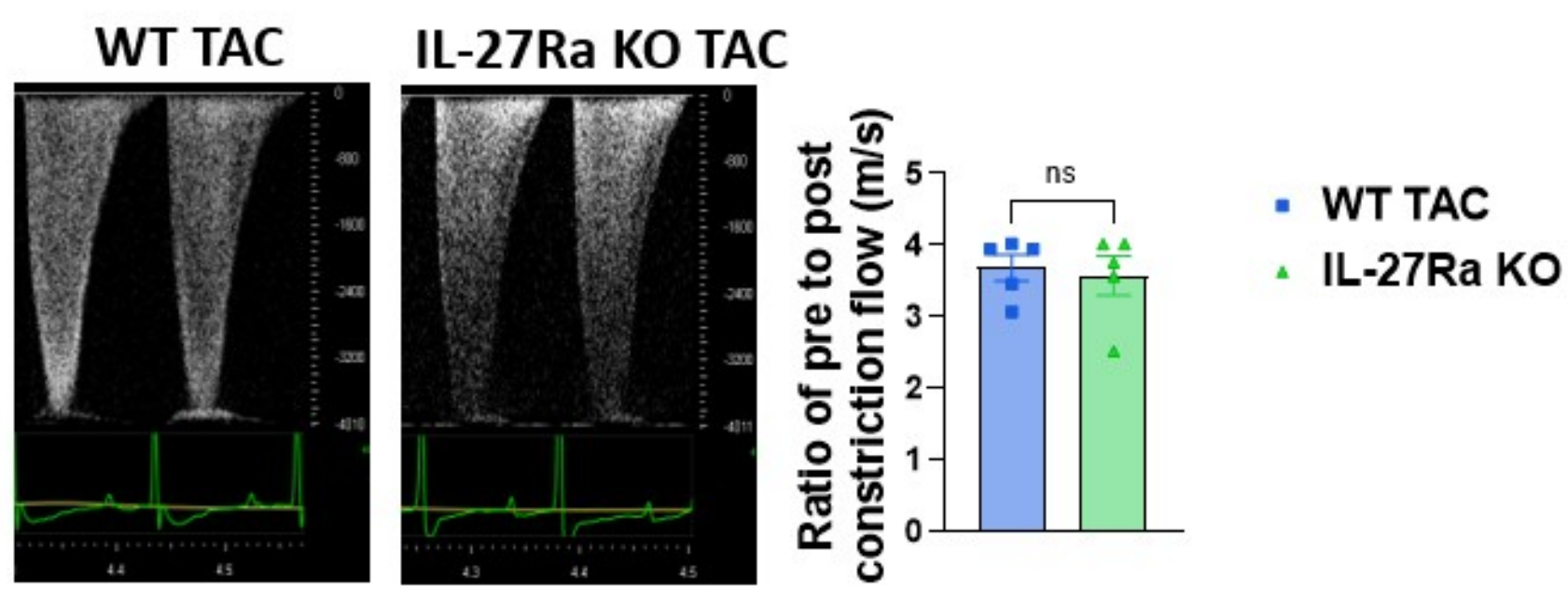

Supplemental Figure 2: Transverse aortic constriction (TAC) induces comparable pressure gradients in wild-type (WT) and IL-27Ra knockout (KO) mice. Representative Doppler echocardiograms of aortic blood flow velocity following TAC surgery. The bar graph quantifies the ratio of pre- to post-constriction flow velocity, confirming no significant difference (ns) in the degree of surgical constriction between genotypes. *n = 5 per group. Data are presented as mean ± SEM.*

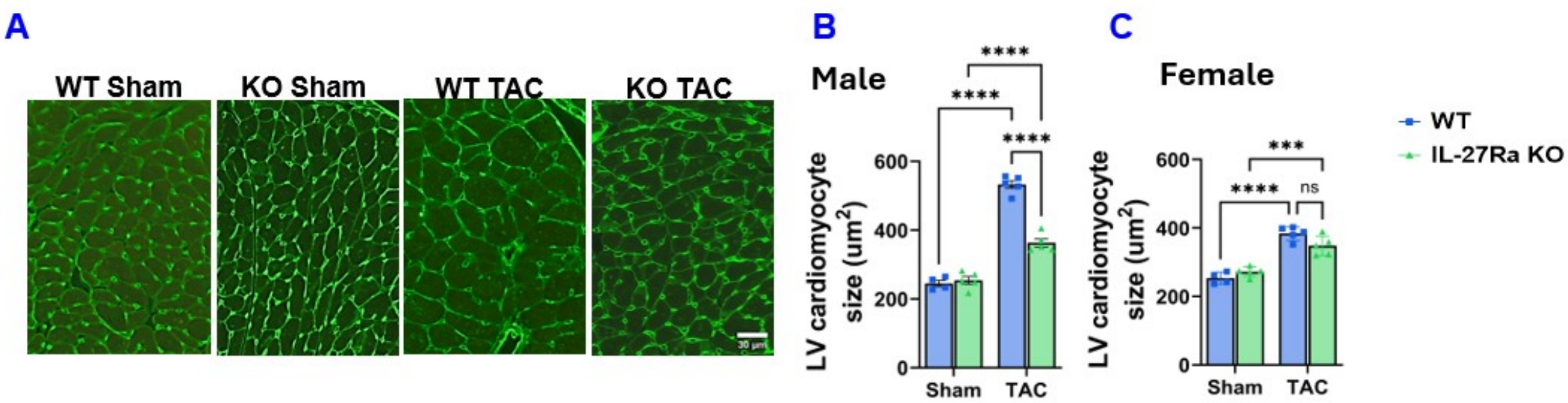

Supplemental Figure 3. IL-27Ra deficiency attenuates TAC-induced cardiomyocyte hypertrophy in a sex-dependent manner. (A) Representative wheat germ agglutinin (WGA) staining of left ventricular myocardial sections to delineate cell membranes. Scale bar = 30 µm. **(B, C)** Quantitative analysis of left ventricular cardiomyocyte cross-sectional area in male **(B)** and female **(C)** wild-type (WT, blue) and IL-27Ra knockout (KO, green) mice following Sham or TAC surgery. n= 4-5 per group. *Data are presented as mean ± SEM. *** $p < 0.001$ or **** $p < 0.0001$; ns = not significant.*

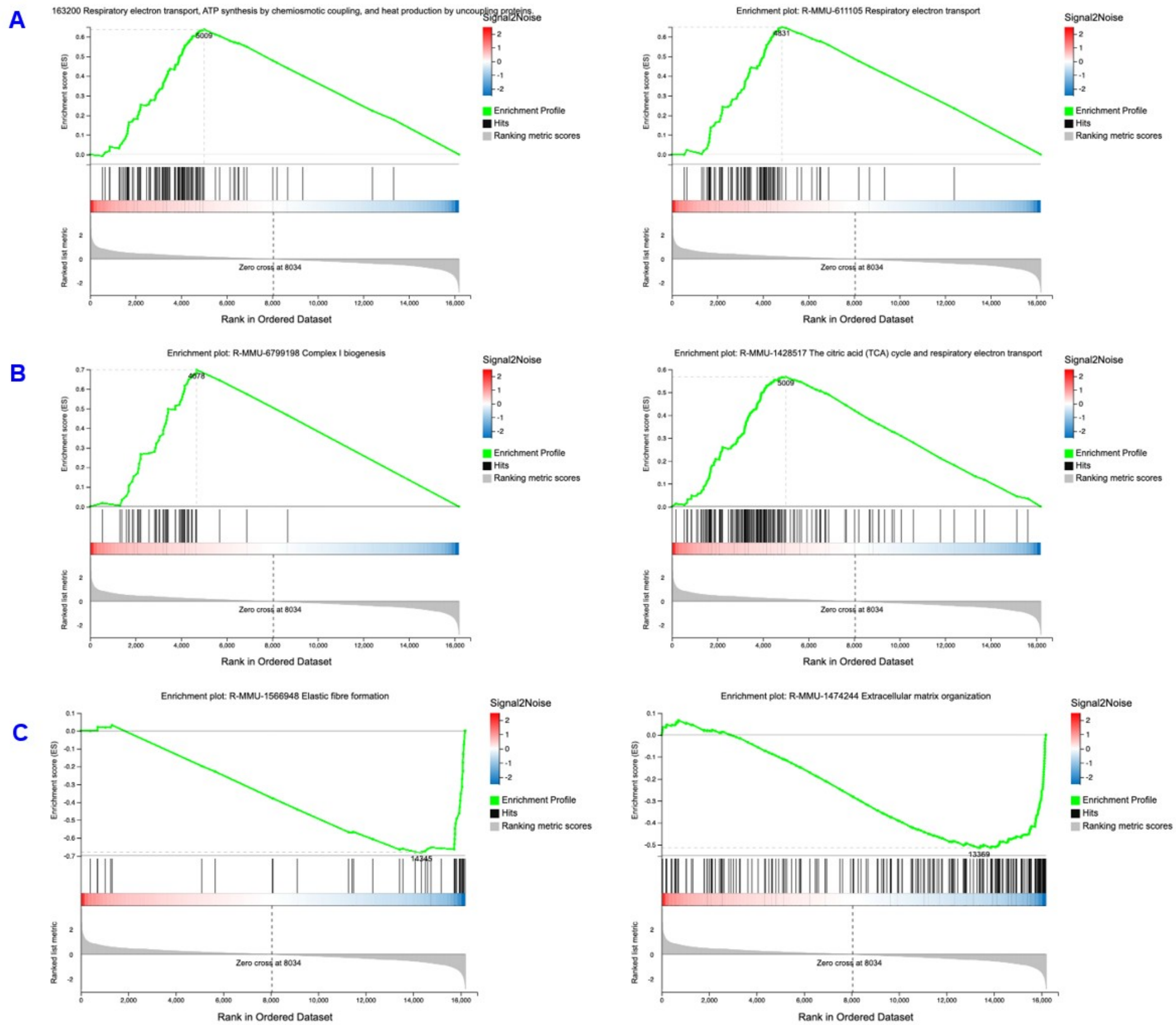

Supplemental Figure 4: IL-27Ra deficiency preserves mitochondrial metabolism and suppresses extracellular matrix remodeling pathways following TAC. Representative Gene Set Enrichment Analysis (GSEA) plots of the left ventricular transcriptome from IL-27Ra KO mice subjected to TAC. **(A, B)** GSEA enrichment plots demonstrating a significant positive enrichment score (ES) for key metabolic and mitochondrial gene sets, including **(A)** Respiratory electron transport and ATP synthesis, and **(B)** Complex I biogenesis and the citric acid (TCA) cycle. The clustering of hits to the left (green profile) indicates substantial upregulation of these energetic pathways in the IL-27Ra KO cohort. **(C)** GSEA enrichment plots displaying a significant negative enrichment for gene sets associated with structural and fibrotic remodeling, specifically Elastic fibre formation (left) and Extracellular matrix organization (right), highlighting the suppression of these pathological networks in the absence of IL-27 receptor signaling.

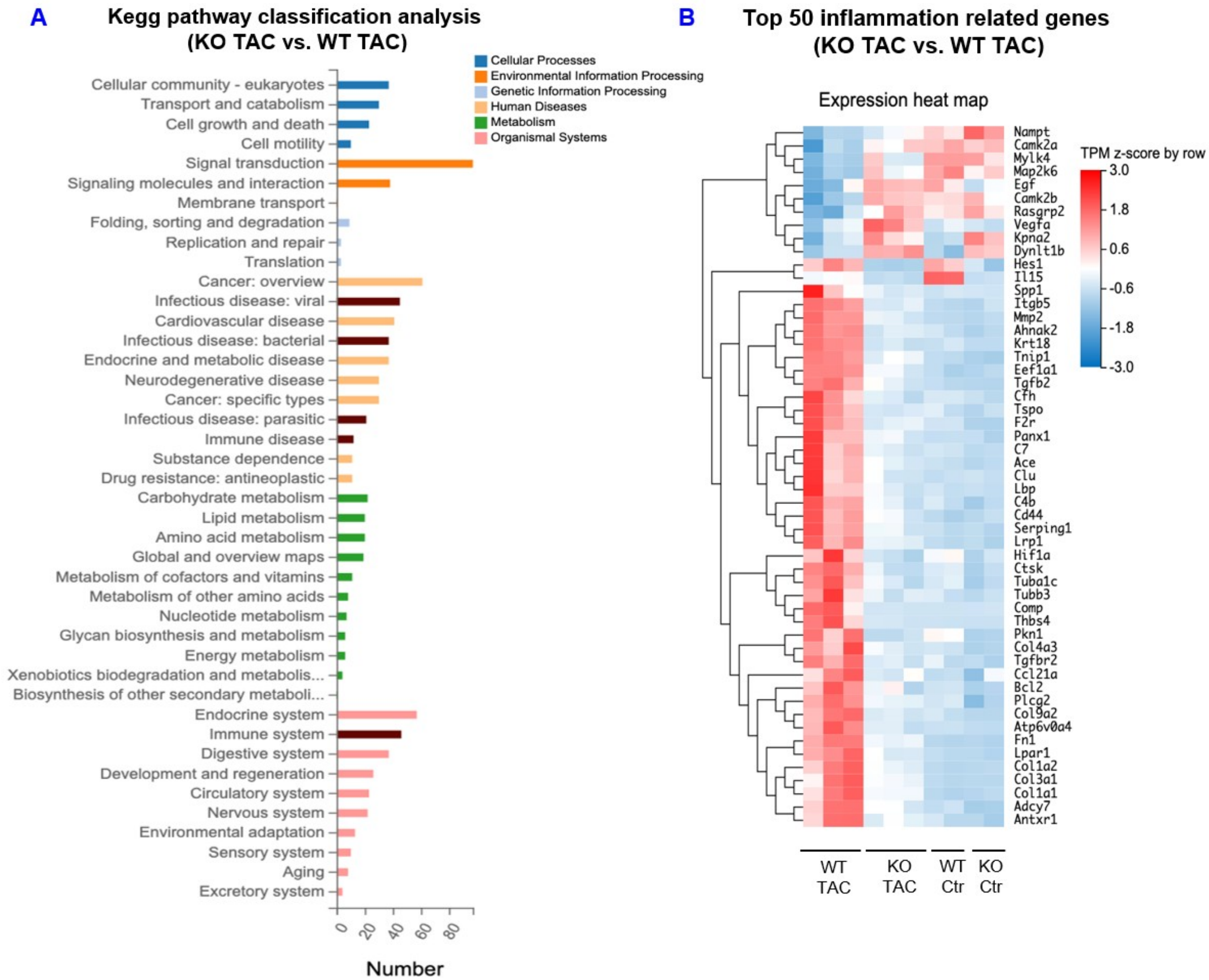

Supplemental Figure 5: Transcriptomic profiling reveals significant upregulation of immune and inflammatory signaling networks. (A) Functional classification of differentially expressed genes categorized by broader biological processes. The analysis highlights a prominent enrichment of genes associated with "Human Diseases" (including viral, bacterial, and parasitic infectious diseases) and "Organismal Systems" (predominantly the immune system). (B) Hierarchical clustering and expression heatmap of the top differentially expressed genes. The color gradient represents the TPM z-score by row, with red indicating relative upregulation and blue indicating downregulation. A distinct module of highly upregulated transcripts includes key mediators of the complement cascade, leukocyte adhesion, and innate immune activation.

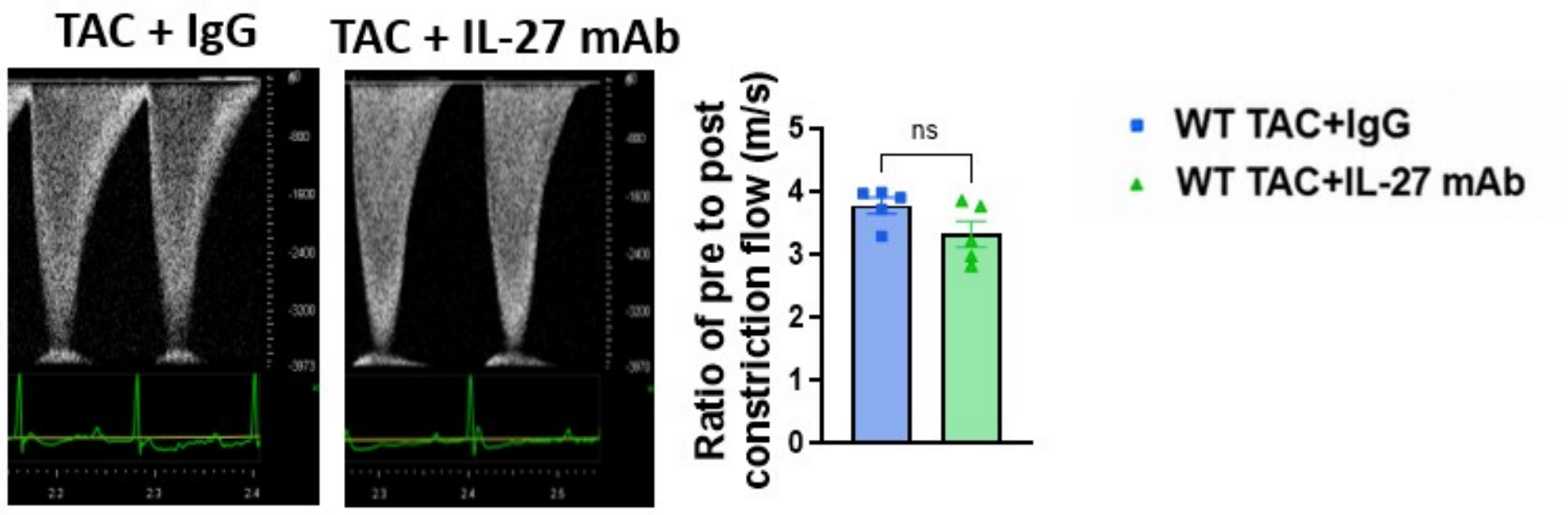

Supplemental Figure 6: Transverse aortic constriction (TAC) induces comparable pressure gradients in mice treated with IgG or IL-27 mAb. Representative Doppler echocardiograms of aortic blood flow velocity following TAC surgery. The bar graph quantifies the ratio of pre- to post-constriction flow velocity, confirming no significant difference (ns) in the degree of surgical constriction between the treatment groups. n = 5 per group. Data are presented as mean ± SEM.

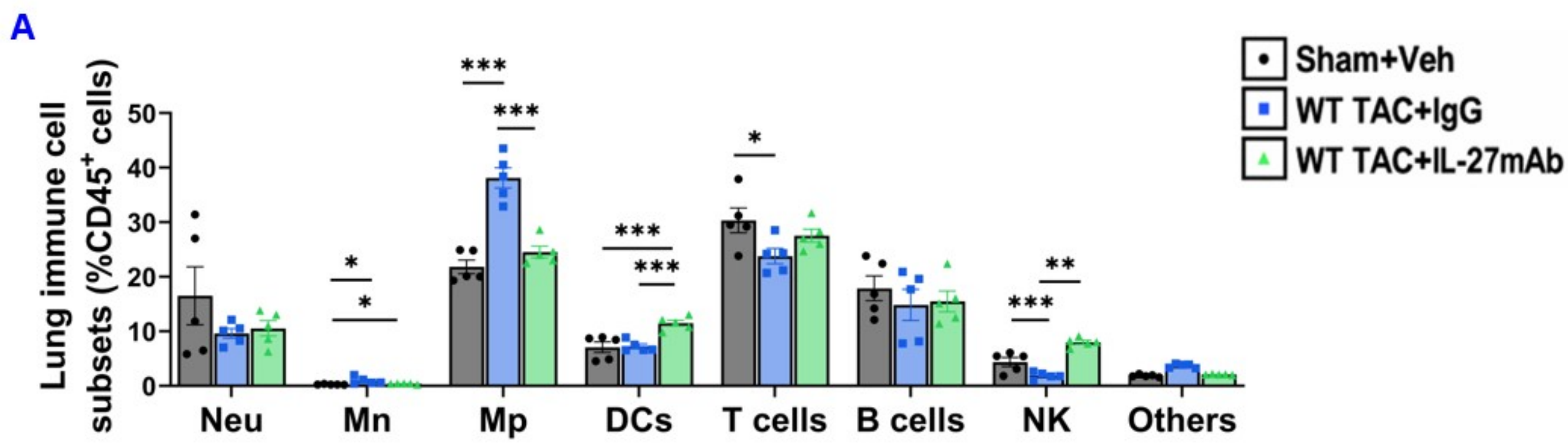

Supplemental Figure 7. IL-27 neutralizing antibody attenuates TAC-induced secondary pulmonary inflammation and macrophage accumulation. Flow cytometric profiling of immune cell populations in the lung tissues of male mice following Sham surgery or transverse aortic constriction (TAC), treated with either an IgG vehicle control or an IL-27 monoclonal antibody (IL-27 mAb). **(A)** Quantitative bar graphs illustrating the relative proportional distribution of major immune cell subpopulations—including neutrophils (Neu), monocytes (Mn), macrophages (Mp), dendritic cells (DCs), T cells, B cells, natural killer (NK) cells, and others—across the three experimental groups. Data are presented as mean ± SEM (n = 5–6 mice/group). *P<0.05, **P<0.01, and ***P<0.001 vs. indicated groups (one-way ANOVA with *Bonferroni* post-hoc test).

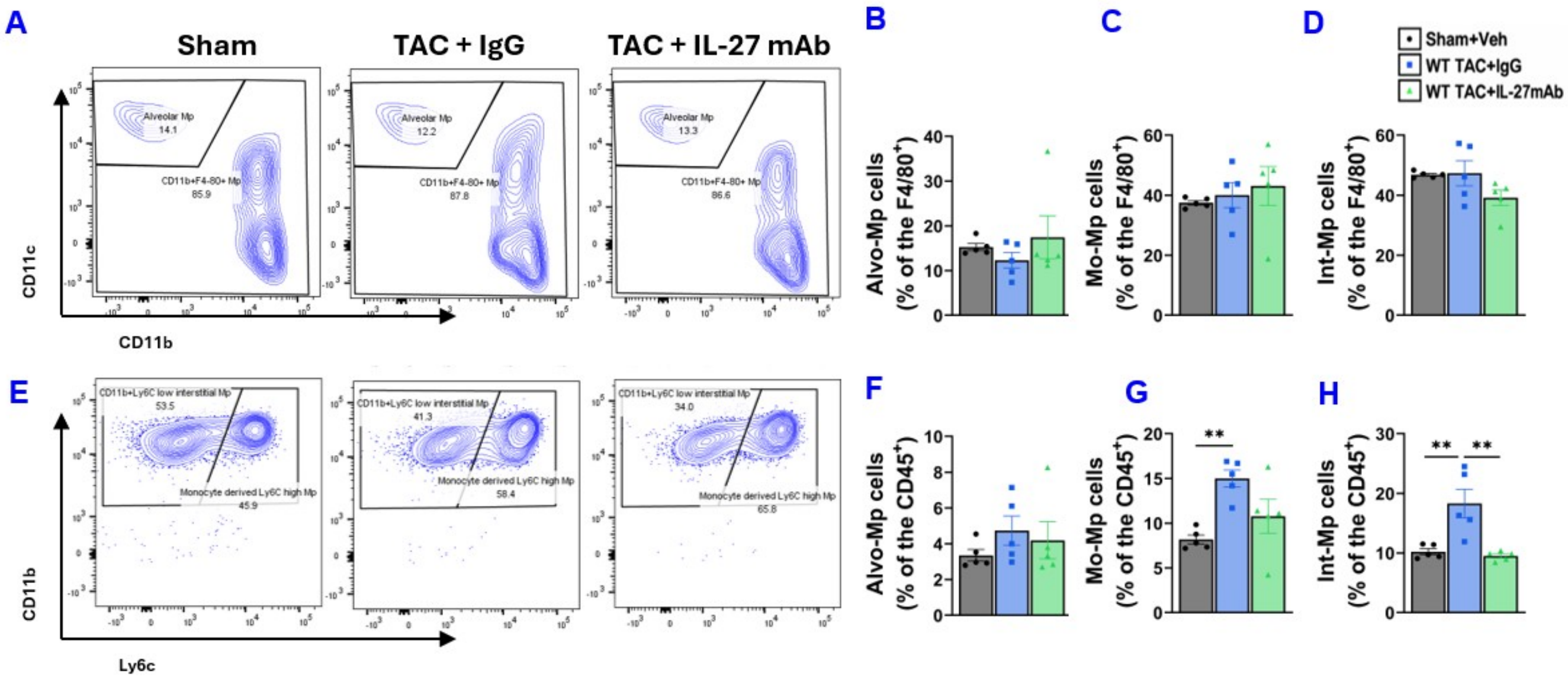

Supplemental Figure 8. Effect of IL-27 neutralizing antibody on TAC-induced secondary pulmonary macrophage subsets (A-H). Representative flow cytometry contour plots (left) and summary bar graphs showing the percentage of alveolar macrophages (Alvo-Mp), interstitial macrophages (Int-Mp), and monocyte-derived macrophages (Mo-Mp) inside pulmonary F4/80+ macrophages **(A-D)** or CD45+ leukocytes **(E-H)** in Sham, transverse aortic constriction with control IgG (TAC+IgG), and TAC treated with an IL-27 neutralizing antibody (TAC+IL27 mAb). *Data are presented as mean +/- SEM (n = 5-6 mice/group). *P < 0.05, **P < 0.01, ***P < 0.001, ****P < 0.0001 vs. indicated groups.*

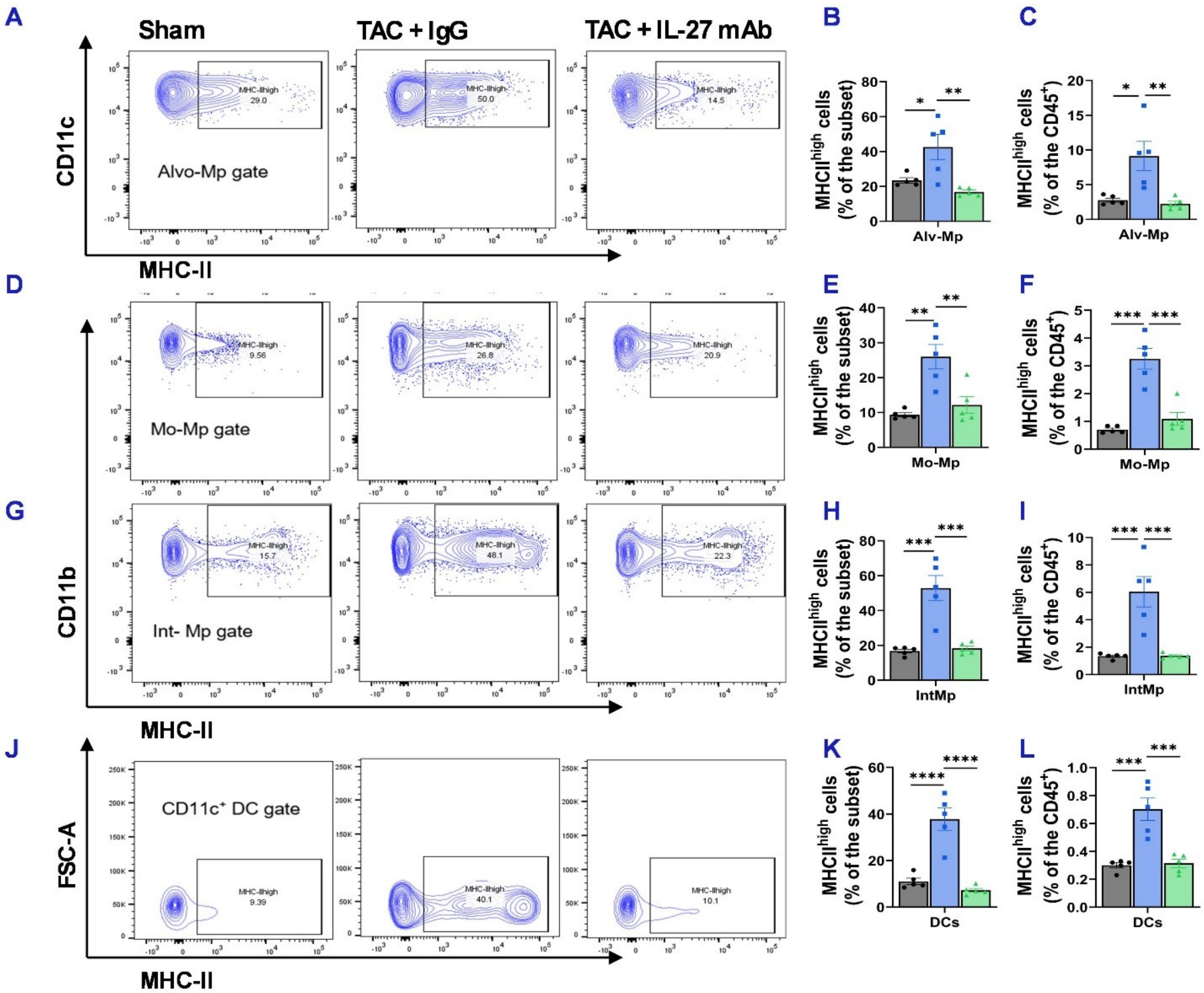

Supplemental Figure 9. IL-27 neutralization attenuates pressure overload-induced upregulation of MHCII in pulmonary myeloid populations. Representative flow cytometry contour plots **(A,D,G,J)** and summary bar graphs showing the percentage of MHCII^{high} cells within the subset **(B, E, H, K)** and total CD45+ population **(C, F, I, L)** for **(A-C)** Alveolar macrophages (Alvo-Mp), **(D-F)** monocyte-derived macrophages (Mo-Mp), **(G-I)** interstitial macrophages (Int-Mp), and **(J-L)** CD11c+ dendritic cells (DCs) in Sham, transverse aortic constriction with control IgG (TAC+IgG), and TAC treated with an IL-27 neutralizing antibody (TAC+IL27 mAb). *Data are presented as mean +/- SEM (n = 4-5 mice/group). *P < 0.05, **P < 0.01, ***P < 0.001, ****P < 0.0001 vs. indicated groups.*

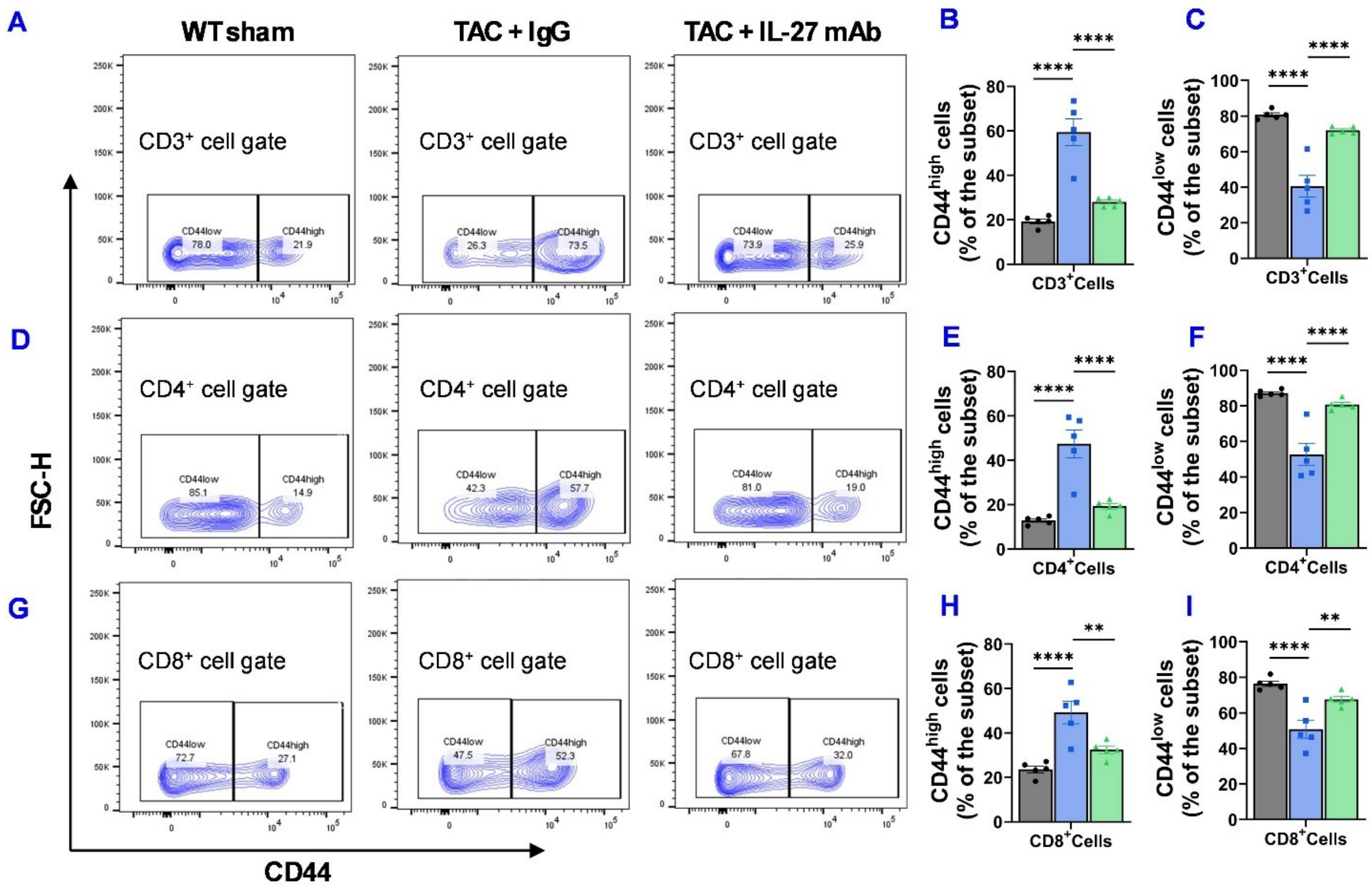

Supplemental Figure 10. IL-27 neutralization suppresses TAC-induced T cell activation in the lung. Quantitative flow cytometric analysis of T cell activation states within lung tissue isolated from Sham (grey), TAC+IgG (blue), and TAC+IL27 mAb (green) cohorts. Bar graphs display the relative percentages of activated ($CD44^{high}$, left column) and resting/naive ($CD44^{low}$, right column) cells within the total T cell (CD3+), helper T cell (CD4+), and cytotoxic T cell (CD8+) populations. Sham+Veh, n = 5; WT TAC+IgG, n = 5; WT TAC+IL-27 mAb, n = 5. *Data are presented as mean ± SEM. Statistical significance is denoted by **p < 0.01 and **** p < 0.0001.*

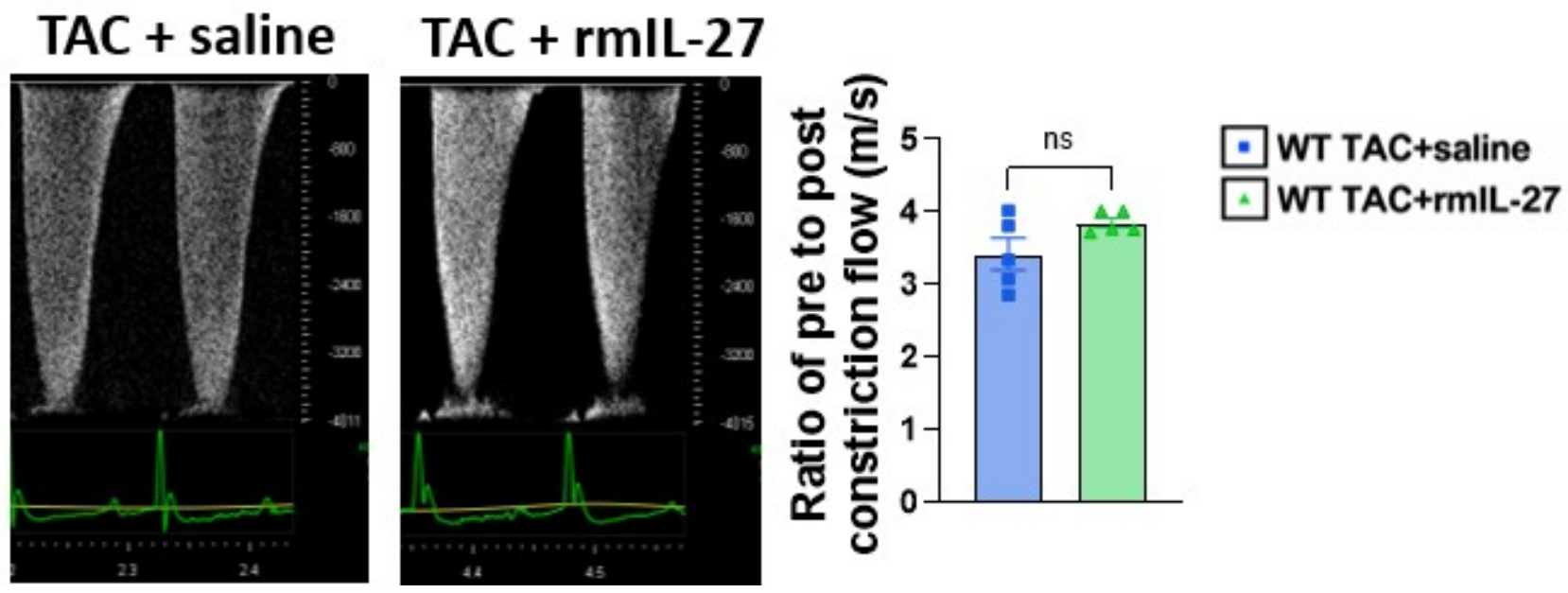

Supplemental Figure 11: Transverse aortic constriction (TAC) induces comparable pressure gradients in mice treated with saline or rmIL-27. Representative Doppler echocardiograms of aortic blood flow velocity following TAC surgery. The bar graph quantifies the ratio of pre- to post-constriction flow velocity, confirming no significant difference (ns) in the degree of surgical constriction between the treatment groups. *n = 5 per group. Data are presented as mean ± SEM.*

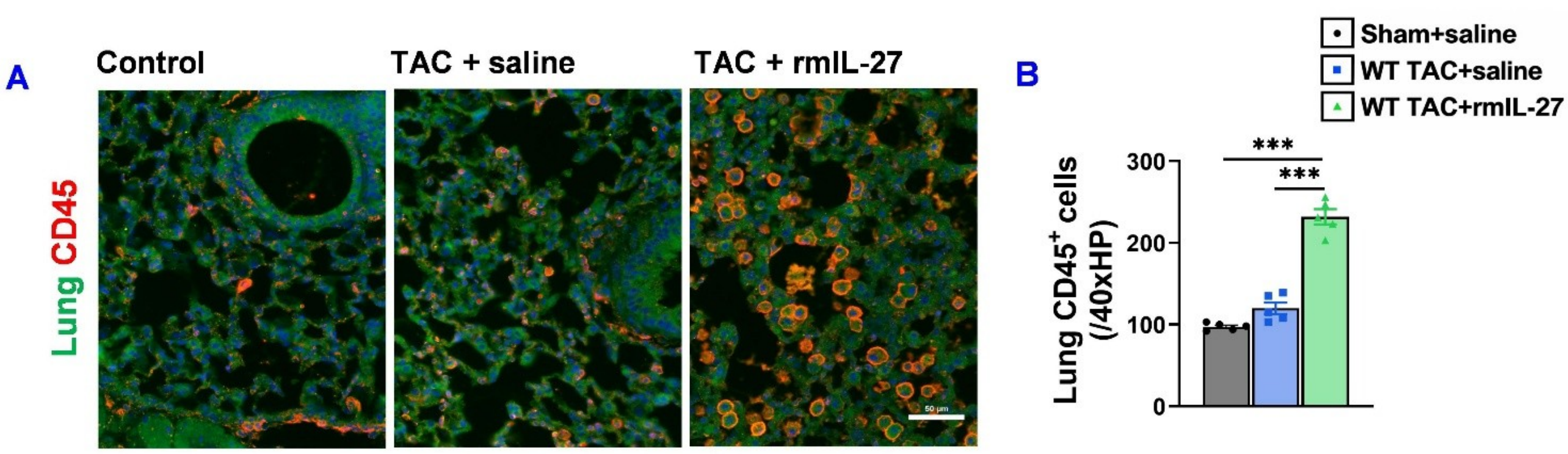

Supplemental Figure 12. rmIL27 increases CD45+ immune cell infiltration in the lung of wild type mice after TAC. (A) Representative immunofluorescence images of lung sections stained for CD45 (red) from Sham, TAC + Saline, and TAC + rmIL27 groups (scale bar: 50 µm). **(B)** Quantification of CD45+ cells per field. *Data are presented as mean ± SEM (n=5). *p < 0.05 vs. TAC + Saline.*

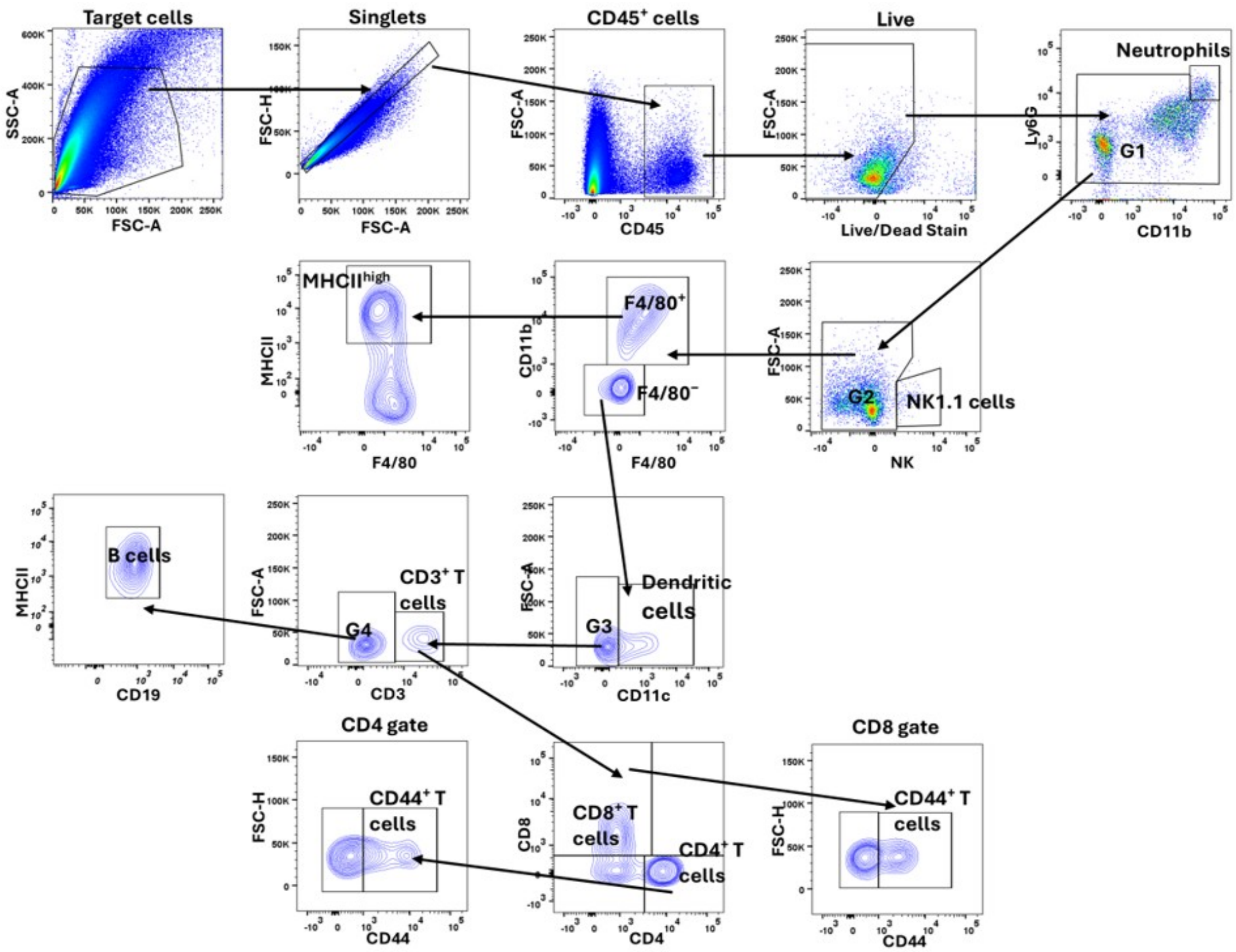

Supplementary Figure 13: Flow cytometry gating strategy used for the identification of cardiac neutrophils, macrophages, dendritic cells, NK1.1^{+} cells, B cells, and T-cell subsets in cardiac tissue. MHC-II^{high} expression was assessed in macrophages and dendritic cells, whereas CD44^{high} expression was assessed in T cell subsets.

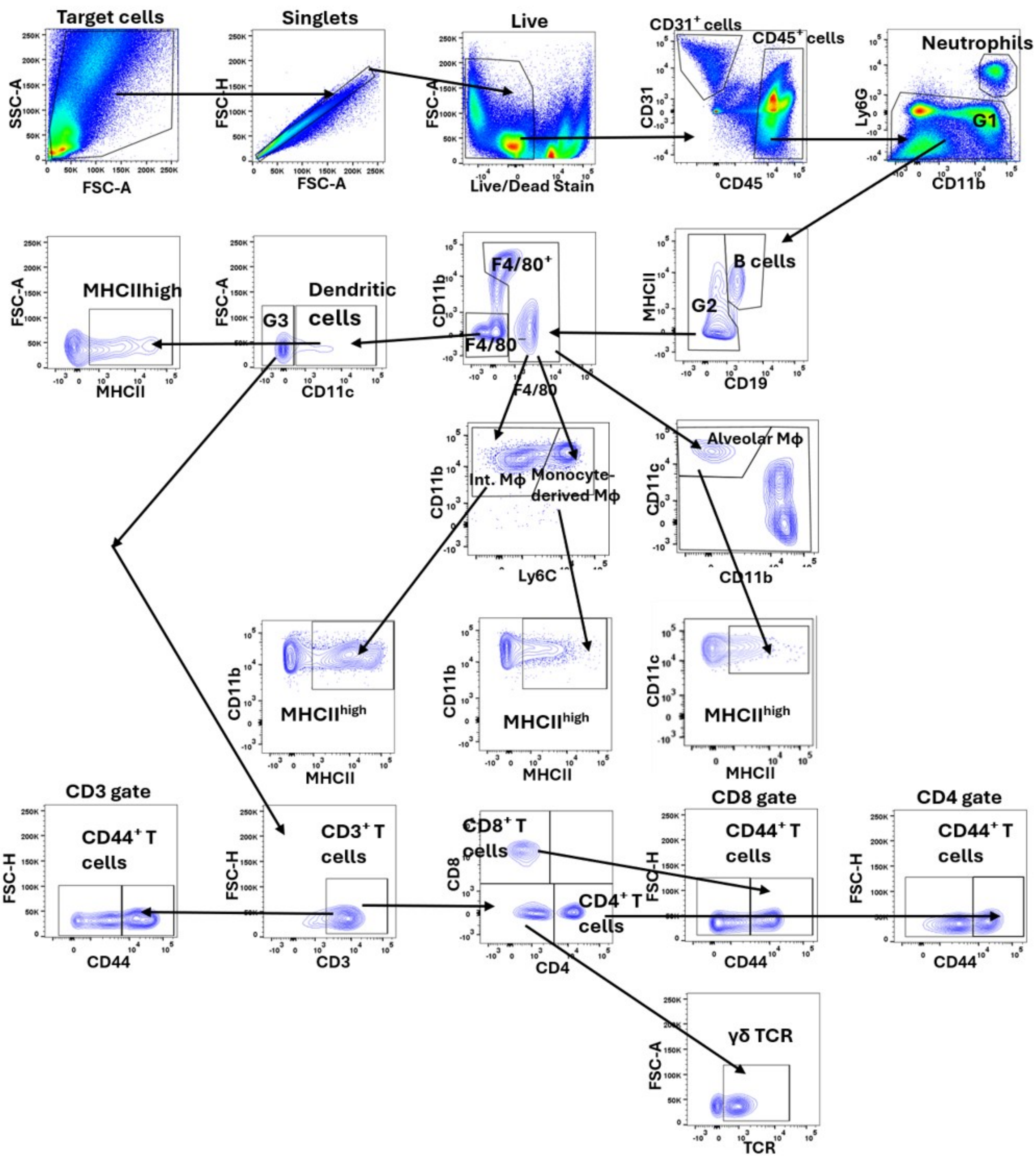

Supplementary Figure 14. Flow cytometry gating strategy used for the identification of pulmonary neutrophils, macrophages, macrophage subsets, dendritic cells, NK1.1^{+} cells, B cells, and T-cell subsets. MHC-II^{high} expression was assessed in macrophages and dendritic cells, whereas CD44^{high} expression was assessed in T-cell subsets, respectively
