# Supplementary material for: IL-27 promotes systolic overload-induced cardiac oxidative stress, inflammation, fibrosis, and heart failure development
Source: Redox Biol. 2026 Oct 28;98:104459. doi: 10.1016/j.redox.2026.104459 (PMC13760151; doi:10.1016/j.redox.2026.104459)
Supplement: Supplementary file 2 [file mmc2.pdf]

Table S11. The top cardiac Deferentially Expressed Genes (DEGs) between IL27Ra KO TAC and /WT TAC mice (|Log2|>1.0 fold).

Gene ID	Gene Symbol	log2 (IL27Ra_KO_TAC/WT_TAC)	Q value (IL27Ra_KO_TAC/WT_TAC)	Average normalized read count KO_TAC	Average normalized read count WT_TAC
57262	'Retnla'	4.000166593	1.79E-04	25.28398309	1.860334529
13088	'Cyp2b10'	2.888707201	0.002275505	23.26126444	3.720669059
69368	'Wdfy1'	2.478251171	1.70E-36	585.5770484	104.1787336
216616	'Efemp1'	2.39331739	1.74E-08	82.93146454	15.8128435
104158	'Ces1d'	2.19908387	0.04874297	1439.164318	313.4663682
245026	'Col6a6'	1.99567972	0.010570436	75.85194928	19.53351256
240595	'Kcnv2'	1.804656906	4.33E-05	162.8288511	46.50836324
19341	'Rab4a'	1.737784638	5.34E-10	147.6584613	43.71786144
26897	'Acot1'	1.677276485	0.001524424	102.1472917	31.625687
381836	'Sbk2'	1.653862858	0.032032752	34.38621701	11.16200718
237403	'Lingo3'	1.635960815	0.010018886	113.2722443	36.27652332
230163	'Aldob'	1.582133116	0.038641524	34.38621701	11.16200718
26399	'Map2k6'	1.481415251	0.003979303	91.02233913	32.55585426
18639	'Pfkfb1'	1.390092578	0.010038152	62.70427807	24.18434888
22295	'Cdh23'	1.362358404	0.046668379	123.3858375	48.36869776
272381	'Lrrc4b'	1.318654469	0.018518907	159.7947731	64.18154126
20519	'Slc22a3'	1.291310646	0.00522964	108.2154476	43.71786144
668940	'Myh7b'	1.2703777	8.62E-04	971.9163101	401.8322584
268902	'Robo2'	1.245053405	0.024249496	84.95418319	36.27652332
11832	'Aqp7'	1.244175678	0.00587201	308.4645937	130.2234171

13195	'Ddc'	1.243691292	0.010673198	69.78379333	28.83518521
13643	'Efnb3'	1.241962901	3.51E-06	1159.017785	487.4076467
53883	'Celsr2'	1.226422367	0.006957141	120.3517595	51.15919956
319953	'Ttll1'	1.195007435	6.94E-09	676.5993875	293.0026884
114663	'Impa2'	1.17794632	5.45E-04	164.8515698	72.55304665
73750	'Whrn'	1.159759836	1.70E-05	349.930326	155.3379332
109050	'Inka2'	1.157956856	5.77E-05	596.702001	266.958005
546611	'Klhl33'	1.144587867	1.34E-05	233.6240038	105.1089009
100042856	'Gvin2'	1.144050776	9.41E-04	1167.10866	528.3350064
27981	'Rsrp1'	1.141981817	4.33E-05	1410.846257	637.1645763
235611	'Plxnb1'	1.128502203	0.012002243	688.7356994	314.3965355
20439	'Siah2'	1.119757007	0.04076473	186.0901156	85.57538835
18115	'Nnt'	1.115464655	6.87E-15	16987.80256	7736.201141
12323	'Camk2b'	1.103317935	2.42E-04	175.9765223	81.85471929
238564	'Mylk4'	1.102414176	6.18E-04	3285.906443	1528.264816
Gm4070	'Gm4070'	1.067754417	1.27E-06	1418.937131	671.5807651
14585	'Gfra1'	1.064832517	4.33E-05	157.7720545	74.41338118
268859	'Rbfox1'	1.064428351	1.07E-04	600.7474383	286.4915175
13645	'Egf'	1.058146765	0.002873841	170.9197257	81.85471929
18784	'Pla2g5'	1.045021838	9.08E-07	269.0215801	129.2932498
216188	'Aldh1l2'	1.031294451	0.030301485	117.3176815	56.74020315
21648	'Dynlt1b'	1.024722636	1.32E-06	264.9761428	129.2932498
12842	'Col1a1'	-1.009306498	1.52E-06	2075.309332	4156.917506
20377	'Sfrp1'	-1.011999578	3.22E-07	249.805753	499.4998211

12825	'Col3a1'	-1.012129441	2.17E-04	4800.92271	9666.298215
11513	'Adcy7'	-1.012148432	1.41E-04	228.5672072	457.6422942
12268	'C4b'	-1.014716253	6.61E-04	193.1696308	382.2987458
14118	'Fbn1'	-1.014737016	1.21E-13	1379.494118	2754.225271
14396	'Gabra3'	-1.02256593	0.029478286	41.46573227	82.78488656
20720	'Serpine2'	-1.030763465	1.07E-04	312.510031	626.9327364
77794	'Adamtsl2'	-1.047377833	5.20E-06	500.6228652	1023.183991
12843	'Col1a2'	-1.05386478	2.03E-08	2701.340754	5576.352752
68585	'Rtn4'	-1.062217287	6.00E-05	1973.162041	4048.087936
18605	'Enpp1'	-1.068590592	0.002352513	132.4880714	278.1200121
11421	'Ace'	-1.075962112	9.00E-04	1014.393402	2093.806513
12257	'Tspo'	-1.076639755	1.36E-05	286.2146886	591.5863804
18162	'Npr3'	-1.085241607	5.29E-04	249.805753	519.0333337
234267	'Gpm6a'	-1.086897406	0.038329884	29.32942039	60.46087221
171580	'Mical1'	-1.108858513	0.006084976	151.7038986	319.9775391
67896	'Ccdc80'	-1.110020529	5.28E-08	1189.358565	2534.705796
14187	'Akr1b8'	-1.116554591	0.003864325	44.49981024	95.80722826
17285	'Meox1'	-1.119337674	2.68E-05	348.9189667	756.2259862
72709	'C1qtnf6'	-1.122851336	1.99E-04	145.6357426	310.6758664
70101	'Cyp4f16'	-1.126297059	0.022135075	32.36349836	68.83237759
14314	'Fstl1'	-1.133980802	1.48E-12	2016.650491	4388.529155
54352	'Irx5'	-1.146289189	0.008373863	71.80651198	157.1982677
14190	'Fgl2'	-1.165339046	6.49E-06	974.950388	2165.429392
252837	'Ackr4'	-1.180500029	0.001476148	72.81787131	161.8491041

11815	'Apod'	-1.183076504	0.016924282	107.2040883	237.1926525
216725	'Adamts2'	-1.18536702	5.54E-15	421.736838	946.9102755
21808	'Tgfb2'	-1.187624364	6.59E-06	265.9875021	598.0975512
16373	'Irx3'	-1.194465591	8.08E-05	220.4763326	494.8489848
100910	'Chpf2'	-1.20624125	0.001020936	129.4539934	291.1423539
17390	'Mmp2'	-1.207568317	1.23E-11	1050.802337	2391.460038
12111	'Bgn'	-1.208998005	7.65E-20	5082.080602	11614.06847
11988	'Slc7a2'	-1.209366705	0.003609789	51.57932551	117.2010754
268857	'Nlrc3'	-1.21261853	9.58E-04	106.192729	243.7038234
16560	'Kif1a'	-1.228648799	0.006738027	26.29534242	61.39103947
242109	'Zfp697'	-1.244605798	0.019255735	109.226807	253.005496
16425	'Itih2'	-1.250879415	0.028636908	23.26126444	54.87986862
19242	'Ptn'	-1.25251933	0.042404588	76.8633086	184.1731184
269855	'Ssc5d'	-1.254787436	2.82E-07	146.6471019	345.0920552
17965	'Nbl1'	-1.260240148	1.93E-04	273.0670174	638.0947436
64817	'Svep1'	-1.262517918	2.31E-12	389.3733396	927.3767629
23886	'Gdf15'	-1.265098693	0.023136019	66.74971536	158.128435
100041194	'Ahnak2'	-1.277376984	4.84E-14	341.8394514	814.8265239
217430	'Pqlc3'	-1.283748596	5.26E-07	63.71563739	153.4775987
58861	'Cysltr1'	-1.311165356	0.018308108	43.48845092	106.0390682
12828	'Col4a3'	-1.311380947	9.51E-05	82.93146454	202.7764637
330723	'Htra4'	-1.318533797	0.023369241	49.55660686	121.8519117
17313	'Mgp'	-1.320578139	4.24E-08	2065.195739	5093.595942
12628	'Cfh'	-1.33407925	6.17E-06	2431.307814	6026.553708

59095	'Fxyd6'	-1.339499335	2.26E-18	369.1461531	924.5862611
104156	'Etv5'	-1.339617658	2.03E-08	194.1809901	481.8266431
214403	'Gm4788'	-1.340202283	0.007924683	41.46573227	103.2485664
107581	'Col16a1'	-1.350687103	1.44E-11	179.0106003	452.0612906
16012	'Igfbp6'	-1.353802385	2.43E-08	180.0219596	452.9914579
20512	'Slc1a3'	-1.355636434	0.0341173	28.31806106	72.55304665
12832	'Col5a2'	-1.376508049	4.02E-21	714.0196825	1832.429511
20317	'Serpinf1'	-1.385864895	1.73E-20	460.1684923	1189.683932
13038	'Ctsk'	-1.388848613	1.05E-06	90.01097981	231.6116489
242702	'Myom3'	-1.402317181	0.02384439	50.56796618	130.2234171
16803	'Lbp'	-1.421784169	0.00179473	95.06777643	247.4244924
83433	'Trem2'	-1.425378504	0.02227509	18.20446783	46.50836324
27047	'Omd'	-1.455045276	0.026937104	16.18174918	41.85752691
72293	'Nkd2'	-1.461346782	0.002824019	43.48845092	117.2010754
109828	'C7'	-1.483752145	0.00319494	33.37485768	90.22622468
76137	'Mcur1'	-1.486494563	4.12E-23	453.088977	1250.144804
55991	'Panx1'	-1.48752153	0.006089128	31.35213903	85.57538835
84094	'Plvap'	-1.503135379	1.82E-04	81.92010522	228.8211471
83691	'Crispld1'	-1.503380085	6.08E-06	37.42029498	105.1089009
66039	'Tmem254a'	-1.509160858	8.90E-11	60.68155942	171.1507767
74116	'Pi16'	-1.5232466	3.48E-14	961.8027168	2718.878915
211623	'Plac9a'	-1.527230159	6.34E-21	165.8629291	470.6646359
319520	'Dusp4'	-1.536904649	0.024179211	22.24990512	63.251374
242316	'Gdf6'	-1.540118078	0.015453716	18.20446783	51.15919956

13448	'Dok1'	-1.55798569	0.004147396	32.36349836	93.01672647
16975	'Lrp8'	-1.561983452	0.002256217	37.42029498	109.7597372
17095	'Lyl1'	-1.567422509	0.003671494	21.2385458	60.46087221
380839	'Serpinb1c'	-1.569423866	0.027368297	34.38621701	99.52789732
238988	'Erc2'	-1.598860484	0.03203079	13.14767121	39.06702512
20378	'Frzb'	-1.613189982	2.99E-11	116.3063222	350.6730588
56312	'Nupr1'	-1.634985075	3.60E-04	66.74971536	204.6367982
12837	'Col8a1'	-1.646615122	4.21E-18	688.7356994	2132.873538
53972	'Ngef'	-1.680451181	0.013661225	14.15903053	42.78769418
140781	'Myh7'	-1.689948138	0.025570641	35395.55361	111448.921
13717	'Eln'	-1.755538409	9.87E-09	470.2820855	1547.798328
22152	'Tubb3'	-1.758232362	0.005263856	19.21582715	64.18154126
16950	'Loxl3'	-1.7627181	2.56E-08	76.8633086	255.7959978
223272	'Itgbl1'	-1.807652213	6.46E-11	190.1355529	653.9075871
71111	'Gpr39'	-1.814519847	0.026199394	9.102233913	29.76535247
67824	'Nmral1'	-1.834162454	0.022318513	19.21582715	65.11170853
667742	'Piezo2'	-1.835131118	0.026019973	11.12495256	37.20669059
20319	'Sfrp2'	-1.837462088	0.005837857	37.42029498	133.9440861
381413	'Gpr176'	-1.874624472	0.028612755	7.079515266	26.04468341
50530	'Mfap5'	-1.885067383	1.48E-12	435.8958685	1582.214517
210622	'Pamr1'	-1.888757342	3.05E-11	74.84058995	274.3993431
68797	'Pdgfrl'	-1.939356782	1.17E-07	40.45437295	154.4077659
21857	'Timp1'	-1.962438558	7.84E-06	41.46573227	159.0586023
22223	'Uchl1'	-1.993441257	0.01017249	154.7379765	599.0277185

16854	'Lgals3'	-2.034124793	2.03E-04	50.56796618	199.9859619
76293	'Mfap4'	-2.071165099	2.06E-28	329.7031395	1375.717384
18383	'Tnfrsf11b'	-2.090979153	0.03160291	6.068155942	23.25418162
330941	'AI593442'	-2.113123138	0.023948108	19.21582715	79.99438476
12840	'Col9a2'	-2.120480273	0.001566082	8.09087459	33.48602153
50781	'Dkk3'	-2.16784865	7.80E-12	102.1472917	450.2009561
16168	'Il15'	-2.209494158	2.02E-10	25.28398309	113.4804063
245038	'Dclk3'	-2.31093068	0.004846413	7.079515266	31.625687
67606	'Fibin'	-2.312680156	9.96E-16	196.2037088	965.5136208
16668	'Krt18'	-2.329246606	3.35E-06	11.12495256	55.81003588
18481	'Pak3'	-2.367483736	0.01230841	12.13631188	59.53070494
329872	'Frem1'	-2.38529218	0.001176522	9.102233913	46.50836324
72275	'2200002D01Rik'	-2.397529428	0.012931319	6.068155942	31.625687
69700	'Col22a1'	-2.434892186	4.95E-05	15.17038986	79.99438476
14402	'Gabrb3'	-2.506528715	0.042226269	3.034077971	15.8128435
78896	'Ecrg4'	-2.55810099	0.009103322	5.056796618	26.04468341
50706	'Postn'	-2.66124485	1.28E-27	1177.222253	7330.648213
20845	'Star'	-2.727597415	0.019850843	33.37485768	212.0781364
22403	'Ccn5'	-2.74401285	8.37E-14	76.8633086	505.0808247
140494	'Atp6v0a4'	-2.850543486	1.20E-06	10.11359324	73.48321391
208164	'Fam180a'	-2.860058148	1.70E-04	5.056796618	31.625687
50490	'Nox4'	-2.878558101	1.66E-10	26.29534242	184.1731184
100033459	'Ifi208'	-2.915008188	1.32E-05	6.068155942	40.92735965
78593	'Nrip3'	-2.917626685	0.021578334	5.056796618	34.41618879

243755	'Slc13a4'	-2.958260693	7.26E-04	4.045437295	28.83518521
20750	'Spp1'	-3.061318666	6.36E-04	8.09087459	63.251374
11752	'Anxa8'	-3.073952822	1.36E-04	7.079515266	57.67037041
230099	'Car9'	-3.138218056	0.019576511	2.022718647	14.88267624
12931	'Crlf1'	-3.184696614	0.001283323	25.28398309	218.5893072
13371	'Dio2'	-3.191381185	1.02E-21	50.56796618	453.9216252
330406	'B4galnt3'	-3.252790754	0.045422211	2.022718647	13.02234171
329941	'Col8a2'	-3.312872629	3.88E-12	18.20446783	178.5921148
218476	'Gcnt4'	-3.441827843	0.027225812	2.022718647	14.88267624
214425	'Cilp'	-3.450871705	1.94E-52	423.7595566	4588.515117
16948	'Lox'	-3.583779181	7.88E-06	58.65884077	674.3712669
12816	'Col12a1'	-3.648938772	3.67E-25	27.30670174	332.0697135
21828	'Thbs4'	-3.697737173	7.44E-12	137.544868	1765.457468
20753	'Sprr1a'	-3.919948127	1.97E-08	5.056796618	70.69271212
16997	'Ltbp2'	-4.003133694	5.44E-12	96.07913575	1519.893311
14264	'Fmod'	-4.04844477	3.94E-09	33.37485768	547.8685189
68588	'Cthrc1'	-4.318400768	2.04E-05	2.022718647	33.48602153
12716	'Ckmt1'	-4.366457793	0.012125903	1.011359324	20.46367982
12845	'Comp'	-4.462765069	7.71E-12	51.57932551	1116.200718
654812	'Angptl7'	-4.640868437	2.38E-04	17.1931085	422.2959382
27083	'Xlr4b'	-5.488617512	0.006248166	1.011359324	16.74301076
12654	'Chil1'	-5.502243122	0.002332346	1.011359324	30.69551974
64103	'Tnmd'	-6.202419627	7.80E-04	1.011359324	26.97485068
246048	'Chodl'	-6.410493877	5.75E-04	0	15.8128435

12814	'Col11a1'	-6.941640964	5.06E-04	0	23.25418162
170942	'Erdr1'	-10.11830303	3.08E-14	0	203.706631
